# Zebrafish Models for Human Skeletal Disorders

**DOI:** 10.3389/fgene.2021.675331

**Published:** 2021-08-05

**Authors:** Manuel Marí-Beffa, Ana B. Mesa-Román, Ivan Duran

**Affiliations:** ^1^Department of Cell Biology, Genetics and Physiology, Faculty of Sciences, University of Málaga, IBIMA, Málaga, Spain; ^2^Networking Biomedical Research Center in Bioengineering, Biomaterials and Nanomedicine (CIBER-BBN), Andalusian Centre for Nanomedicine and Biotechnology-BIONAND, Málaga, Spain

**Keywords:** skeletal dysplasia, osteogenesis imperfecta, osteoporosis, skeletal ciliopathies, dwarfisms, dysostosis, osteopetrosis, zebrafish models

## Abstract

In 2019, the Nosology Committee of the International Skeletal Dysplasia Society provided an updated version of the Nosology and Classification of Genetic Skeletal Disorders. This is a reference list of recognized diseases in humans and their causal genes published to help clinician diagnosis and scientific research advances. Complementary to mammalian models, zebrafish has emerged as an interesting species to evaluate chemical treatments against these human skeletal disorders. Due to its versatility and the low cost of experiments, more than 80 models are currently available. In this article, we review the state-of-art of this “aquarium to bedside” approach describing the models according to the list provided by the Nosology Committee. With this, we intend to stimulate research in the appropriate direction to efficiently meet the actual needs of clinicians under the scope of the Nosology Committee.

## Introduction

Skeletal disorders are a heterogeneous group of rare hereditary diseases with many different skeletal symptoms and molecular mechanisms of disease (Krakow and Rimoin, 2010). These diseases are characterized by skeletal defects that appear during development and/or growth, the dysplasia, or at late stages and/or adulthood. These disorders may also show symptoms in other organs. Although skeletal disorders are considered rare diseases, they affect around 1.5% of births and emerge as a primary scientific objective in modern countries. Due to the available data that arise from whole genome (Wheway et al., 2015) or exome (Bamshad et al., 2011) sequencing of patients and genome-wide association studies (GWASs) (Kemp et al., 2017), sufficient information is being screened for the genotype/phenotype characterization of many of these diseases.

During the last 50 years, these disorders have been subjected to a continuous revision by a Nosology Committee of the International Skeletal Dysplasia Society. This committee periodically provides a classification of them according to anatomical or physiological symptoms and genomic data, the Nosology and Classification of Genetic Skeletal Disorders (NCGSD). In the last version (Mortier et al., 2019), 461 skeletal diseases were classified in 42 groups, and 437 genes were assigned as causative of 425 of them. The anatomical features used to classify these diseases range from the tissue (chondrodysplasia or osteodysplasia) or cell affected (i.e., osteoclasts dysfunction in osteopetrosis), the severity of phenotype (achondroplasia vs. hypochondroplasia), specific genotype/phenotype relationships (FGFR3 chondrodysplasia and collagenopathies), cell functions affected (i.e., ciliopathies or cohesinopathies), or even the specific cell pathologies involved (i.e., ER-cell stress). In the NCGSD-2019, single pathogenic gene variants may be associated with several disease groups, and various phenotypes in turn may be assigned to more than one gene mutation. This list aims to help a correct diagnosis of each disease and to challenge the research of effective therapies (Mortier et al., 2019).

To conduct experiments or drug tests against these diseases, human cell culture assays and animal models have been developed in different species including mice, rat, or zebrafish. These models are also used to analyze the genetic basis of disease and the nature of the mutant genes found in human probands. Encouraged by its easy handling, body transparency of embryos, and the wide range of genetic and transgenic variants in many labs around the world, zebrafish is increasingly offering a relevant alternative to rodent models. This review is focused on the zebrafish models for human skeletal disorders found in the bibliography available at PubMed, ZFIN, or Google Scholar (see section “Data Collection Method”).

For some skeletal diseases (i.e., NCGSD group 25), reviews have been published that compare models in various species and zebrafish (Enderli et al., 2016; Luderman et al., 2017; Kwon et al., 2019). In general, mice strains are the models of preference to study the basis of disease or to search for treatments. The human genome is 80–90% identical to that of mice (Yue et al., 2014), but only 71% to the zebrafish genome (Howe et al., 2013). Furthermore, whereas mice and human genomes have suffered the same number of polyploidization events, many of zebrafish genes are duplicated when compared with humans, suggesting an additional genome duplication (Amores et al., 1998). This may complicate the identification of ortholog gene functions (Kwon et al., 2019). Also, zebrafish phenotypes can be only partially compared with human defects, as certain skeleton features vary in composition or morphogenetic processes (Mork and Crump, 2015; Luderman et al., 2017).

Nevertheless, zebrafish is increasingly used to evaluate chemical treatments (Tomecka et al., 2019) or to search for new genetic models (i.e., Gistelinck et al., 2018). Whereas new studies on drug dosage, mechanical loading, or long-term effects of treatments may still use mice (Enderli et al., 2016), zebrafish is becoming crucial at the *in vitro* to *in vivo* transition in drug screening pipelines (rev. Enderli et al., 2016; Luderman et al., 2017; Carnovali et al., 2019, see below). Zebrafish is also a choice to confirm gene discovery, to perform genetic analysis of human variants, or to study orthologs to mouse lethal alleles. In the latter case, zebrafish gene duplicates may be functionally balanced by sub-functionalization (Force et al., 1999), so that viable loss of function (LOF) alleles (alleles without function) of a specific paralog can be obtained and studied (Kwon et al., 2019; Busse et al., 2020). Either way, when a skeletal disorder gene is duplicated in zebrafish, a preliminary study is required to successfully generate the model. These technical benefits provided by zebrafish models and their relevance at these initial stages of pre-clinical studies may be at the heart of a new aquarium-to-bedside transference revolution (Santoriello and Zon, 2012).

This transference requires a better knowledge of the models on the part of clinicians and translational researchers, and sometimes the refocusing of zebrafish scientists to the actual clinical needs. Many reviews have recently been published to address the use of zebrafish models of skeletal disorders. In these reviews, genetic (Eisen and Smith, 2008; Auer and Del Bene, 2014; Kwon et al., 2019; Tonelli et al., 2020a; Dietrich et al., 2021) and phenotyping (Kwon et al., 2019; Busse et al., 2020; Tonelli et al., 2020a) methods and many of available models have been described (Luderman et al., 2017; Carnovali et al., 2019; Kwon et al., 2019; Busse et al., 2020; Tonelli et al., 2020a; Dietrich et al., 2021). Nevertheless, no review has updated this rapidly growing field under the directives of the NCGSD. While 20 (Luderman et al., 2017) and 61 (Tonelli et al., 2020a) models have been described in the most extensive reviews, only three (Luderman et al., 2017) and 25 (Tonelli et al., 2020a) of these models genetically approach the human diseases annotated in the NCGSD. In our review, we classify 90 zebrafish models according to the NCGSD and compare phenotypes and genetic perturbations in humans and zebrafish to provide appropriate information to the skeletal genetic disorder community. A big data resource of integrated genomics, DanioNet (Shim et al., 2016), has also been obtained. This resource studies co-functional relationships among zebrafish genes for *function-driven* disease gene discovery. This resource highly predicts zebrafish genes and heterozygous rare variants in proband orthologs for one of the groups proposed by the NCGSD, the ciliopathies group. This resource opens new venues for validation of whole-exome sequencing data (Shim et al., 2016).

## Zebrafish Models for Human Skeletal Diseases

The popularity of *Danio rerio* as a research model for vertebrate studies is certainly rooted in the thousands of genetic or transgenic variants, many of them available in repository labs [Zebrafish International Resource Center (ZIRC), European Zebrafish Resource Center (EZRC), or China Zebrafish Resource Center (CZRC)]. This species has been included in drug screening pipelines for many human diseases (rev. Santoriello and Zon, 2012; García-Caballero et al., 2018; Varga et al., 2018; Truong and Artinger, 2021) and its husbandry conditions standardized to respect their natural conditions (Engeszer et al., 2007; Arunachalam et al., 2013). Although not present in many international directives, a consensus on animal care has almost been reached (Geisler et al., 2016; Lawrence, 2016; Varga et al., 2016; Lidster et al., 2017; Aleström et al., 2020), which is becoming crucial for its worldwide pre-clinical use.

In principle, zebrafish is a model of choice for chemical assays that evaluate drugs against bone fragility symptoms. These assays are conducted by the addition of chemical reagents to system water or embryo medium and a subsequent phenotypic study. Osteoporotic drug testing in zebrafish started by the need to study the osteoporotic effects of glucocorticoids in patients under immunosuppressive treatments. The hormonal response of zebrafish to glucocorticoids mimics that shown by humans, providing an interesting non-genetic model for bone formation, mineralization, and osteoclast activity (Dietrich et al., 2021). Once dexamethasone or prednisolone is added to water, osteoporosis is induced in zebrafish, and potential preventing drugs can be tested (Dietrich et al., 2021). Fluorescent calcein staining has also been used to test calcification in wild-type strains in standard conditions or during bone fracture modeling (Bae et al., 2017; Son et al., 2019; Tomecka et al., 2019). At this point, it is necessary to acknowledge other studies that screen anti- or pro-osteogenic drugs (de Vrieze et al., 2015; Chen et al., 2017). In them, calcein labeling (Chen et al., 2017) or an *ex vivo* sp7:luciferase zebrafish scale assay (de Vrieze et al., 2015) have been used. The first study has identified two pro-osteogenic and six anti-osteogenic compounds, whereas the second evaluated 77 previously characterized compounds with correct prediction for 70%. Other references have also tested the pro-osteogenic effects of chemical or extracts, such as DMP-PYT (Bae et al., 2017) or Logan fruit extract (Son et al., 2019), using calcein labeling. These chemical assays may provide a gold standard to be reached by alternative genetic assays.

A variety of techniques are used to modify orthologous genes in zebrafish. Ethyl-*N*-nitrosourea (ENU) or insertional mutagenesis, transgenesis, and morpholino knockdown at the one-cell stage and CRISPR/Cas9 or TALEN editing of genes are used to obtain models. The phenotype of each model is studied by different methods at both the anatomical and cellular levels and compared with human symptoms to clarify its potential utility. The nature of the mutations found in the probands can also be studied by comparing the phenotype of mutant animals with that shown by zebrafish that overexpress exogenous wild-type and/or mutant human or zebrafish genes after injection. This analysis helps to gain insights into the mechanisms of disease.

Several advantages and disadvantages are found when genetic techniques are considered. Alongside classical mutagenesis or transgenesis approaches, morpholino knockdown has become popular for their low cost and quick, genome-wide application against gene translation, pre-mRNA splicing, or miRNA activity. Nevertheless, morpholinos require specific methods to evaluate its effectiveness and to reduce off-target effects (Eisen and Smith, 2008). Some of these control methods are also becoming popular when used in zebrafish skeletal models. These methods are negative (missense) control morpholino knockdown, comparison of morphant and LOF mutant phenotypes, and morphant phenotype rescue by co-injection of mRNA or cDNA (see below).

The great potential provided by morpholinos has preceded a new trend to model diseases by gene editing, techniques that target site-specific insertions anywhere in the genome. Although zinc-finger nucleases (ZFINs) have been successfully used in zebrafish (Doyon et al., 2008; Meng et al., 2008; Gaj et al., 2013), TALEN (Sander et al., 2011) and CRISPR-Cas9 (Liu et al., 2017; Wu N. et al., 2019) are the choice to edit genes causing skeletal disorders (Auer and Del Bene, 2014). These techniques have further advantages when compared with morpholino knockdown, as they produce less off-target effects, and their outcomes are genetically stable individuals.

Furthermore, comparison of zebrafish phenotypes and human symptoms requires a detailed anatomical and histological description. X-ray imaging, micro-computed Tomography, and bone histology are some of these techniques. Several tissue-specific transgenic lines with marker gene promoters that drive fluorescent proteins in bone, cartilage, or blood vessels are also available to perform this task (Tonelli et al., 2020a). These methods provide pharmacological researchers with enough genetic and phenotyping tools for pre-clinical studies.

In Supplementary Table 1, we show the zebrafish models of skeletal genetic diseases currently available. We follow the NCGSD (Mortier et al., 2019) to classify phenotypes, genetic alterations, and references. A total of 84 zebrafish models can be found for 78 human diseases in 27 of the 42 groups. While only one model has been proposed for eight of these groups (groups 1, 3, 14, 18, 19, 30, 31, and 35), several models have been suggested for the 19 remaining groups. Of them, groups 9 (ciliopathies with major skeletal involvement), 25 (osteogenesis imperfecta (OI) and decreased bone density), 34 (dysostoses with predominant craniofacial involvement), 39 (limb hypoplasia-reduction defects), and 41 (polydactyly–syndactyly–triphalangism) show more than five models.

In principle, more than 60 knockdown or gene editing models have been published (Supplementary Table 1), 50 of which have not been reviewed previously (i.e., Tonelli et al., 2020a). Although the genes affected in these models only represent 18% of the total of NCGSD genes, they emphasize the rapid emergence of zebrafish models during the last 10 years. To simplify the text, we have cataloged these models according to NCGSD groups (Supplementary Table 1). Examples of drug testing using these models are also listed in Supplementary Table 2. Unexplored zebrafish mutant strains or genetic perturbations (i.e., *TRPV4* mutants for group 8) are also cataloged for further development in Supplementary Table 3. Previous comparative studies among human, rodent, or zebrafish genes associated with diseases in some of these groups conclude conserved modules of bone- or ciliopathy-related genes in vertebrates (Shim et al., 2016; Kwon et al., 2019). Our review agrees with this conclusion extending it to other NCGSD groups.

### *FGFR3* Chondrodysplasias

Group 1 is characterized by mutations in *FGFR3* gene, which produce over-activation of the pathway. This causes several dwarfism-related syndromes that include the lethal thanatophoric dysplasia types 1 and 2 (Yamashita et al., 2014; Ornitz and Legeai-Mallet, 2017), achondroplasia, hypochondroplasia, and related syndromes. A mutation in zebrafish *fgfr3* gene mimicking one of these genetic aberrations also induces the overexpression of Fgfr3 downstream genes and dorsalized embryos (Lindy et al., 2016). Although this is a good example of the molecular approach provided by these studies, the early embryonic lethality shown by this mutant (Supplementary Table 1) precludes its direct use for drug testing by phenotypic rescue at the larval or adult stages.

### Type XI Collagenopathies

Group 3 is characterized by mutations in collagen type 11. Among other diseases, patients with mutations in this gene may suffer from early osteoarthritis or Stickler syndrome type 2 or 3, with the latter being characterized by distinctive facial features, eye disorders, hearing loss, and joint hypermobility. A nonsense mutation in zebrafish *col11a2* gene has been elegantly described by Lawrence et al. (2018), which shows altered jaw and joint development as well as several skeletal and cellular craniofacial abnormalities. This leads to both impaired jaw function and premature adult osteoarthritis (Supplementary Table 1), which closely resemble the symptoms of Stickler syndrome type 3 in humans.

### Skeletal Ciliopathies

Eighteen different zebrafish models have been proposed for six of the 13 human diseases in group 9 (ciliopathies with major skeletal involvement). Some of these models show both embryonic and larval or adult phenotypes with close similarity with human skeletal symptoms and cell culture abnormalities (Supplementary Table 1). This group of zebrafish models clearly approaches a gold standard for pre-clinical trials due to the variety of assays offered for genetic testing and pharmacological pipelines.

Diseases such as short rib polydactyly syndrome (SRPS), Jeune asphyxiating thoracic dysplasia, cranioectodermal dysplasia, and Mainzer–Saldino syndrome are caused by mutations in a group of 27 genes involved in primary cilia signaling. Of these, 12 genes have been modeled in zebrafish (Supplementary Table 1). These models were obtained by the embryonic injection of morpholinos or RNAs, and ENU or insertional mutations in *ift52*, *ift81*, *kiaa0753*, or *ift172*. Although this group is one of the best covered by zebrafish models, some of the most important genes causing skeletal ciliopathies such as *DYNC2H1* for SRPS are missing. This does not mean that mutants for these genes do not exist, as, for example, at least 6 *dync2h1* mutants are available in the ZFIN repository with point mutations, five of them causing premature termination of the Dync2h1 protein (see below). These and other undescribed zebrafish mutants are waiting for validation and may be invaluable in the search for therapeutic approaches for SRPSs.

Among the 18 zebrafish models for skeletal ciliopathies, several interesting phenotypes relevant to human syndromes have been described, including defects in the otoliths, craniofacial cartilage, and other skeletal elements. Nevertheless, other unrelated developmental phenotypes have also been observed, such as defects during gastrulation or organogenesis. These models have a ventrally curved body axis and various developmental abnormalities in common that affect ectoderm and neuroectoderm formations (hydrocephalus or microphthalmia), heart or blood vessel development (pericardial edema, situs inversus, or intracranial hemorrhages), or kidney formation (pronephric cysts), all of which are partial phenocopies of human defects. Mutated or knockdown genes in this group are associated with cilia formation, structural stability, and/or function. Some of them regulate retrograde intra-flagellar transport in two different protein complexes, *IFT-A* (*ift122*, *ift140*, and *wdr19* genes) and *IFT-B* (*ift172*, *ift52*, *ift80*, and *ift81* genes), but others still have no well-defined functions assigned. This latter group includes a t-SNARE mediator in transport vesicle docking (*tctex1d2*), centrosome-associated proteins (*cep120* and *kiaa0753*), or a DNA damage repair protein (*nek1*), but all have been unambiguously associated with the cilia (Supplementary Table 1). These studies may evaluate genetic interactions between causative genes (Halbritter et al., 2013) or even extend the analysis to other genes involved in the generation of symptoms [i.e., *tsc1a* (DiBella et al., 2009); *bbs4* and *bbs8* (Hudak et al., 2010); or *tekt1* (Ryan et al., 2018)].

### Models for Skeletal Dysplasia Affecting Long Bones and/or Vertebrae

Groups 10 to 14 in the NCGSD inform (Mortier et al., 2019) comprise diseases that affect either the epiphysis (groups 10 and 13) or metaphysis (groups 11 to 14) of long bones or the vertebrae (spondylos in Greek, groups 12–14). Mutations in 47 different genes cause 46 syndromic diseases in these groups. Symptoms range from short stature or dwarfism; scoliosis or lordosis; pectus carinatum; cleft palate; osteoarthritis; joint hyperextensibility; leg, hand, or foot deformities; to problems with vision. Special symptoms can be found in the diseases of group 11 such as broad and fragile bones; deformities of the knees, collar bones, ribs, or hand bones; or the delayed eruption and misalignment of the permanent teeth. The mutations that cause these diseases affect genes coding for proteins involved in the formation of the extracellular matrix (ECM) in the bone and cartilage (collagens, non-collagenous structural proteins, or metalloproteinases), gene transcription or translation, or secretory pathway and cell signaling.

Several zebrafish models have been proposed for diseases in these groups. One model in group 14 (*sbds* model for severe spondylodysplastic dysplasias), two models in group 11 (*rmpr* mutant and *sbds* morphants for metaphyseal dysplasias), and four models in group 13 [*ddrgk1*, *extl3*, *nans*, and *tonsl* models for spondylo-epi-(meta)-physeal dysplasias (SE(M)D)] comprise the offer for pre-clinical studies of these groups. In general, these models show craniofacial cartilage deformities at the embryonic and juvenile stages, bone ossification delays, or limb defects. While some models are not completely understood, the molecular and cellular bases of others have been sufficiently studied (Supplementary Table 1).

It is important to acknowledge some limitations of these models. For instance, the severe spondylometaphyseal dysplasia (SMD) (Sedaghatian-like) in group 14 has been associated with lethal human Shwachman–Bodian–Diamond syndrome (SBDS) mutations. Nevertheless, the fetal or neonate symptoms clinically described are significantly different to those observed by morphants grown under the experimental doses used in a study by Provost et al. (2012).

Models for two metaphyseal dysplasias (group 11) are found in the literature. The CRISPR/Cas9 editing of mitochondrial RNA−processing endoribonuclease *rmrp* gene in zebrafish shows overexpression of the Wnt pathway, as in human metaphyseal dysplasia (Sun et al., 2019). These larvae display a type of chondrodysplasia with disorders in the patterning and shaping of pharyngeal arch-derived bones and cartilages. The ceratobranchial arches, pharyngeal cartilage, teeth, and ceratohyal or basihyal cartilages may show defects in this mutant. The phenotype is also associated with delays in the ossification of intramembranous, vertebra, or head bones or the enhancement of this process in endochondral bones. These phenotypic and cellular aspects are reminiscent of cartilage-hair hypoplasia (metaphyseal dysplasia, McKusick type) in group 11.

As for SMD Sedaghatian-like in group 14 (see above), a second model in this group has been proposed for metaphyseal dysplasia with pancreatic insufficiency and cyclic neutropenia (SBDS, in group 11) by downregulating *sbds* gene. The abnormal gill arches and ceratohyal cartilages, inner ear bone, and twisted tail (Provost et al., 2012), as well as the pancreatic deficiency and neutropenia shown by morphants (Venkatasubramani and Mayer, 2008; Provost et al., 2012; Carapito et al., 2017), are reminiscent of the skeletal abnormalities, pancreatic insufficiency, and chronic neutropenia found in these patients (Provost et al., 2012). As stated above, skeletal phenotypes do not exactly mimic human symptoms, but the loss of neutrophils and pancreatic deficiency found in *sbds* morphants has strongly prompted this model (Provost et al., 2012).

Gene overexpression, knockout mutations, knockdown morpholino, or CRISPR/Cas9 modifications in four zebrafish genes (*ddrgk1*, *extl3*, *nans*, and *tonsl*) have also been published to model SE(M)D. These genes are orthologs of those causing four different skeletal diseases in group 13 (*ddrgk1* for SEMD Shohat type, *extl3* for SEMD with immune deficiency *EXTL3* type, *nans* for SEMD with intellectual disability *NANS* type, and *tonsl* for SPONASTRIME Dysplasia; Supplementary Table 1). The phenotypes shown by genetically affected zebrafish resemble the symptoms found in human patients. Whereas gene expression studies are insufficient for a sensible model (*dymeclin*; Denais et al., 2011), the study of the hypomorph *extl3* mutant *boxer* (Volpi et al., 2017), the morpholino downregulation of *ddrgk1* (Egunsola et al., 2017) or *nasna* (van Karnebeek et al., 2016), and the CRISPR/Cas9 editing of *tonsl* (Burrage et al., 2019) could be considered appropriate zebrafish models for these four SE(M)D dysplasias (Supplementary Table 1). Although these models do not exactly reproduce the symptoms found in humans, several molecular and cellular results support their great potential.

The downregulation of zebrafish *ddrgk1* affects cartilage formation. The morphants show craniofacial cartilage deformities in both the embryonic and juvenile stages. This is potentially exerted by the direct inhibition of Sox9a ubiquitination, as suggested by studies in human cells and rescue of the *ddrgk1* MO phenotype by *sox9a* mRNA injection (Egunsola et al., 2017). Defects affecting pectoral fin and branchial arch development in the *extl3* mutant allele *boxer* (Schilling et al., 1996; van Eeden et al., 1996) have also been reported. The mutation in this glycosyltransferase involved in heparan sulfate biosynthesis somehow reproduces SEMD with immune deficiency *EXTL3*-type symptoms (Volpi et al., 2017). The pectoral fin and branchial arch modifications in the mutant could be reminiscent of the increased intervertebral spaces, short and plump limb or digit bones, skull craniosynostosis, or narrowing of the laryngotracheal tract in patients. Furthermore, defective thymopoiesis has also been found with this mutant, which is reminiscent of the immune deficiency found in humans (Volpi et al., 2017). Moreover, the facial dysmorphism found in patients with SEMD with intellectual disorders *NANS* type could be associated with the small head and craniofacial cartilage phenotypes obtained in zebrafish morphants of *nasna* gene (van Karnebeek et al., 2016). Finally, several skeletal phenotypes have been described in zebrafish with CRISPR/Cas9-edited *tonsl* gene that resembles those of SPONASTRIME dysplasia (Burrage et al., 2019). Lethality before 20 days post-fertilization (dpf), accelerated vertebra ossification, short body size, and spinal abnormalities in the mutant resemble the spine abnormalities, disproportionately short stature, and exaggerated lumbar lordosis or scoliosis found in humans with SPONASTRIME dysplasia (Burrage et al., 2019). These results strongly suggest the potential pre-clinical use of these models in the genetic analysis of human mutant alleles or the search for treatments.

### Models for Acromelic and Acromesomelic Dysplasias and Dysostoses

Groups 15–17 of the NCGSD (Mortier et al., 2019) comprise diseases producing dwarfisms by reductions of the fore and/or hind limbs (acromelic, groups 15–16) and hands and/or feet (mesomelic, groups 16–17).

Two zebrafish models for four diseases in group 15 and another two for three dysplasias in group 17 (mesomelic and rhizo-mesomelic dysplasias) have been developed and further studied. No models for diseases in group 16 (acromesomelic dysplasias) have been found in our search.

Mutations in *FBN1* gene have been associated with three diseases in group 15 (geleophysic dysplasia, acromicric dysplasia, and Weill–Marchesani syndrome) and one disease in group 30 (see below) (Mortier et al., 2019). These mutations disorganize Fibrillin-1 in elastin fibers, reducing the strength and flexibility of connective tissue and the control of its growth and repair by the TGF-β stored in the ECM. Besides short stature, symptoms associated with these diseases are reduced long bones in the limbs; short hands, feet, fingers, or toes; specific facial features; heart, liver, lung, or ear defects; or abnormal eyes with myopia (Chen et al., 2006). While the morpholino knockdown of the zebrafish *fbn1* ortholog shows an expanded ventral fin fold with vascular defects that resemble Marfan syndrome in group 30 (see below), the situs inversus or pronephros cyst found in the morphants is reminiscent of symptoms in patients with these acromelic dysplasias (Chen et al., 2006). Another model based on *pde4d* morphants also resembles the symptoms of LOF mutations in human *PDE4D* gene that cause acrodysostosis. In this case, the pathogenicity of specific mutations in humans has been investigated via the overexpression of mutant transcripts in the morpholino background (Lindstrand et al., 2014).

Regarding group 17, morphants of zebrafish *shox* gene also show growth retardation and decreased vertebral and craniofacial ossification (Kenyon et al., 2011; Sawada et al., 2015; Marchini et al., 2016; Montalbano et al., 2016), which resembles symptoms shown in patients with dyschondrosteosis (Leri-Weill) or mesomelic dysplasia Langer type, diseases caused by *SHOX* mutations. Moreover, some skeletal disorders described in human patients with the dominant type of Robinow syndrome may have been mimicked in zebrafish. This disease is caused by different genetic disorders including *WNT5A* hypomorphic alleles. The mutant alleles in humans have been probed in zebrafish by the injection of hypomorphic or dominant negative (DN) human mRNAs into one-cell stage embryos (Person et al., 2010). Cell coalescence in the pancreatic islet, axis duplication, and bent tail phenotype are shown in this model (Person et al., 2010).

### Models for Campomelic Dysplasia

Campomelic dysplasia is a rare disease in group 18 caused by mutations in *SOX 9*. This disorder affects skeletal, face, and external genitalia development. Patients normally show bowed limbs and short legs among other symptoms. Only one model has been proposed for campomelic dysplasia that studies the zebrafish *jellyfish* (*sox9a*) mutant. This mutant shows craniofacial, neurocranium pharyngeal arch, and pectoral girdle cartilage defects (Yan et al., 2002; Nissen et al., 2006; Gordon et al., 2014; Plavicki et al., 2014). These defects, further supported by eGFP or mCherry expression assays, strongly resemble some symptoms shown by campomelic dysplasia patients. These symptoms are dislocated hips, underdeveloped shoulders, special facial features, or a group of physical symptoms associated with neural crest cell disorders called the Pierre–Robin sequence (Chen et al., 2019).

### Models for Primordial Dwarfism

Group 19 classifies disorders with a proportionate size reduction of all bones (primordial dwarfism). Only one zebrafish model has been proposed for this group. The CRISPR/Cas9 editing of zebrafish *cog4* gene has been used to model Saul–Wilson syndrome (Ferreira et al., 2018). These larvae show malformed inner ears, semicircular canals or lateral lines, deafness, short body sizes, craniofacial defects, and abnormal pec fins (Ferreira et al., 2018). These phenotypes grossly resemble the dwarfism, facial features, and abnormal structure of long bones described in patients with this disease in group 19.

### Models for Skeletal Dysplasia With Joint Dislocations

The following group in the NCGSD, group 20, includes disorders with multiple joint dislocations. Of the 15 disorders in this group, only two models have been suggested for human diseases: *slc10a7* morphants for multiple joint dislocations with amelogenesis imperfecta and *fam20b* mutants for severe (lethal) neonatal short limb dysplasia with multiple dislocations.

The morpholino knockdown of zebrafish *slc10a7* gene causes tooth cartilage to be bent downward, whole body edema, craniofacial disorders, and curled body in growing larvae. These phenotypes mimic the skeletal dysplasia with scoliosis, defective tooth enamel formation, and facial dysmorphism shown by patients with multiple joint dislocations with amelogenesis imperfecta (Ashikov et al., 2018).

Two zebrafish *fam20b* mutants, *b1125* and *b1127*, show larval primary chondrocyte and perichondral bone defects that resemble symptoms of severe neonatal short limb dysplasia with multiple dislocations, a lethal human genetic condition caused by mutations in *FAM20B* gene (Eames et al., 2011). These phenotypes are rescued after the injection of *fam20b* cDNA and strongly support its potential use as another model of skeletal dysplasia.

A third study from our group can be found (Durán et al., 2011) that downregulates *plod1a* zebrafish gene, ortholog to the human gene causing Ehlers–Danlos syndrome kyphoscoliotic type 1 in this group (Mortier et al., 2019). The morphant against this *lysyl hydroxylase 1* shows fin fold deformity and ventral body curvature (Supplementary Table 1) that grossly resemble joint laxity and scoliosis, human symptoms of this disease.

### Models for Osteopetrosis and Sclerosing Bone Disorders

In the osteopetrosis and related disorders group (group 23), 11 different diseases are cataloged to be caused by a mutation in 15 different genes (Mortier et al., 2019). Only two zebrafish models of these diseases have been published, the *clcn7* morphant (Zhang et al., 2019) and *csf1r* mutants (Oosterhof et al., 2019). The morpholino downregulation of the *CLCN7* zebrafish ortholog (*clcn7*) leads to Tgf-β-mediated defects in craniofacial cartilage and teeth that mimic some of the symptoms shown by patients with the severe neonatal, infantile, intermediate, or late-onset forms of osteopetrosis (Zhang et al., 2019).

Among osteopetrosis-like disorders, only dysosteosclerosis has been modeled in zebrafish. *CSF1R* is one of the three genes that cause this syndrome, which in zebrafish is duplicated and encoded by the genes *csf1ra* and *csf1rb*. To model dysosteosclerosis, *csf1ra* mutants were obtained by chemical mutagenesis, and *csf1rb* was gene-edited by TALEN. The phenotypes of these larvae include small vertebral arches and a brain microglia deficiency that resembles the symptoms of this disease (Oosterhof et al., 2019).

Two other diseases in group 24 (other sclerosing bone disorders), hyperostosis–hyperphosphatemia syndrome, caused by mutations in *GALNT3* gene, and Lenz–Majewski hyperostotic dysplasia, a result of *PTDSS1* mutations (Mortier et al., 2019), have been modeled in zebrafish. LOF TALEN-edited or ENU-induced *galnt3* mutants in zebrafish show hyperostosis and ectopic calcium deposits (Bergen et al., 2017; Stevenson et al., 2017) that resemble human hyperostosis caused by autosomal recessive mutations in *GALNT3* gene (Mortier et al., 2019). Moreover, the transpose-mediated transgenesis of mutant forms of human *PTDSS1* gene in zebrafish produces mild scoliosis with incomplete penetrance (Seda et al., 2019) mimicking symptoms of Lenz–Majewski hyperostotic dysplasia. No model of disease in group 21 (chondrodysplasia punctata) or 22 (neonatal osteosclerotic dysplasias) has been published.

### Osteogenesis Imperfecta Models

Osteogenesis imperfecta is the most represented dysplasia in zebrafish models and the best example of what these models can be used for. Within the NCGSD group 25 of OI and decreased bone density, there are 17 models for nine different diseases, some of which are actively used in drug screening pipelines in several labs, including ours. Most of the mutations that cause OI in human fall in the genes coding for *COL1A1* and *COL1A2*. Accordingly, zebrafish orthologs *col1a1a*, *col1a1b*, and *col1a2* represent the highest number of genetic modifications in proposed OI models. Besides the collagen mutations, around 20% of cases with OI and related diseases are due to mutations in other non-collagen genes that participate in collagen synthesis or related signaling pathways. Thus, a further 10 human orthologs have been experimentally modified in zebrafish (Supplementary Table 1). In this sense, chemical or CRISPR/Cas9-edited mutants and some morpholino knockdowns of the genes *crtap*, *p3h1* (ortholog to *LEPRE1*), *bmp1b*, and *sparc* have been used to model OI types with mutations in non-collagen genes (Supplementary Table 1).

Mutants and morphants of collagen genes show small and malformed cartilage, severe craniofacial dysmorphology, anomalous and non-healing fractures in fin rays, and bone fragility (Asharani et al., 2012; Durán et al., 2011; Fiedler et al., 2018; Gistelinck et al., 2018). These defects resemble symptoms found in classic OI types 1–4. The CRISPR/Cas9 editing of the zebrafish *crtap* or *p3h1* genes leads to growth delay, short size, head and body disproportion, a deformed spine, calli in ribs, delayed bone mineralization, swim bladder inflation, or high mortality (Tonelli et al., 2020b). This has led authors to propose these mutants as models for OI, or as it is more accepted in the OI community research like models within the OI general spectrum (see Forlino and Marini, 2016; Supplementary Table 1). Morphants and mutants of *bmp1* (Asharani et al., 2012; Cho et al., 2015; Hur et al., 2017; Gistelinck et al., 2018; Tomecka et al., 2019; rev. Enderli et al., 2016) and *sparc* (Kang et al., 2008; Rotllant et al., 2008) genes have also been proposed as models for OI replicating human mutations after finding reduced ossification with low bone density or phenotypes in the otoliths, pharyngeal arches, or inner ear. Mutations in human orthologs to all these genes have been described as causes of different types of OI (Mortier et al., 2019).

Regarding OI-related and decreased bone density disorders, the symptoms observed in patients with mutations in the *PLS3*, *MBTPS2* (X-linked osteoporosis), *PLOD2* (Bruck syndrome type 2), *SEC24D* (Cole–Carpenter like dysplasia), *PYCR1* (Cutis laxa type 2B), or *NBAS* (SOPH syndrome) genes have also been modeled in zebrafish by changes in the corresponding orthologous genes. Morpholino knockdown of the *pls3* (van Dijk et al., 2013: rev. Besio et al., 2019), *mbtps2* (Schlombs et al., 2003), *sec24d* (Sarmah et al., 2010; Garbes et al., 2015), or *nbas* (Palagano et al., 2018) genes serves as models of disease in this group. Also, mutations in *mbtps2* (Schlombs et al., 2003; rev. Besio et al., 2019), *plod2* (Gistelinck et al., 2016; Hur et al., 2017), *sec24d* (Sarmah et al., 2010; Garbes et al., 2015), or the TALEN editing of *pycr1* (Liang et al., 2019) show phenotypes such as bone fragility, sclerosis, growth retardation, or other skeletal dysmorphologies that resemble phenotypes in these syndromes in humans.

Besides these genetic models and given the similarity of OI with non-idiopathic osteoporosis, zebrafish models in this group are sometimes generated chemically using dexamethasone, prednisolone, or prednisone, providing easy experiments in which several drugs are being assayed (Bergen et al., 2019).

### Abnormal Mineralization, Lysosomal Storage, and Osteolysis Disorder Models

In this subsection, we summarize models of a complex set of disorders: rickets (*VDR* type) in group 26, mucopolysaccharidosis (*IDS* type) and mucolipidosis (*GNPTAB* type) in group 27, and mandibuloacral dysplasia and multicentric osteolysis, nodulosis, and arthropathy (MONA) in group 28. The abnormal mineralization in two types of rickets, the aberrant lysosomal storage with skeletal symptoms in mucopolysaccharidosis and mucolipidosis, and the skeletal dysmorphologies of two osteolysis syndromes have also been modeled.

A model for vitamin D-dependent rickets type 1B or 2A is the abnormal ossification of vertebrae in morphants of *vdra* and *vdrb* genes (Lin et al., 2012; Kwon et al., 2019). Group 27 zebrafish models show phenotypes of lysosomal storage diseases (Supplementary Table 1). These are the dysmorphologies generated by the altered migration of neural crest cells in *ids* morphants or CRISPR/Cas9 mutant (Moro et al., 2010; Bellesso et al., 2018) or several phenotypes shown by morphants and TALEN-edited mutants of *gnptab* (Flanagan-Steet et al., 2009, 2016, 2018; Petrey et al., 2012). Finally, the morpholino downregulation of zebrafish *lmna* gene or the transgenic overexpression of mutant human alleles of *LMNA* (Koshimizu et al., 2011) has been proposed as models of mandibuloacral dysplasia, whereas the CRISPR/Cas9 editing of *mmp14* (De Vos et al., 2018) has been proposed as a model of MONA syndrome.

### Models for the Disorganized Development of Skeletal Components

Under the NCGSD group 29, only models of osteochondromas (*EXT2* type) and fibrodysplasia ossificans progressiva (FOP) (*ACVR1* gene) have been investigated in zebrafish. The zebrafish *etx2* (*dackel*) mutant shows cartilage condensation, hypertrophic chondrocytes, and ossification defects as well as an abnormal morphology of the teeth that is rescued by FGF8 bead implants (Clément et al., 2008; Wiweger et al., 2012, 2014). This phenotypic and cellular behavior is reminiscent of multiple cartilaginous exostoses, a group 29 of human diseases caused by autosomal dominant mutations in *EXT2* gene.

Fibrodysplasia ossificans progressiva in humans is caused by a mutation that confers the constitutive activation of the ACVR1 receptor (Shen et al., 2009) and shows heterotopic ossifications (HOs) and malformations of great toes. The transgenic gain of function (GOF) of zebrafish *acvr1l* gene also shows HO lesions, spinal lordosis, vertebral fusions, and malformed pelvic fins, while the LOF mutants in *acvr1l* show loss of ventral tail tissue (Shen et al., 2009; LaBonty et al., 2017; Mucha et al., 2018; LaBonty and Yelick, 2019). This not only supports an FOP model but also is a complementary tool to perform the genetic analysis of FOP mutations in probands. One chemical assay has been combined with this FOP model, the injection of cardiotoxin, a myonecrotic agent that induces non-local HO (LaBonty et al., 2018). In this experiment, spine and body cavity HOs were also obtained after local injuries by caudal fin clips. Mixed models of this type may enhance the potential of both approaches in drug testing.

### Marfan Syndrome Models

For diseases in group 15, we have already studied the overgrowth phenotypes when zebrafish *fbn1* gene is perturbed in morphants (see above). Although the expanded ventral fin fold and situs inversus are weak results when compared with the extensive skeletal and heart symptoms seen in Marfan syndrome, this is a first approach in the search of an appropriate model for diseases in group 30 (Chen et al., 2006).

### Models for Dysplasia Involving Inflammatory/Rheumatoid-Like Osteoarthropathies

One group in the NCGSD (group 31, genetic inflammatory/rheumatoid-like osteoarthropathies) includes five diseases that cause cartilage degeneration, osteomyelitis, periostitis, pustulosis, or fibromatosis with inflammatory etiology. Only one zebrafish model has been suggested for progressive pseudorheumatoid dysplasia (PPRD), a disease that is associated with swollen joints, pain, and the abnormal deposit of calcium as it worsens through life. This disease is caused by autosomal recessive mutations in *WISP3* gene.

The morpholino downregulation of zebrafish *ccn6* gene, an ortholog of *WISP3*, shows mandibular and pharyngeal cartilage size and shape disorders and fragility (Nakamura et al., 2007, Supplementary Table 1). These defects may resemble some of the symptoms shown by patients. No other study using this model has been found in the literature.

### Models for Disorders Involving Skeletal Dysostosis and/or Craniosynostosis

Five groups in the nosology, groups 32–36, comprise 50 human dysplasias that produce skeletal dysostosis or craniosynostosis (Mortier et al., 2019). The disorders in these groups are cleidocranial dysplasia and related disorders (five disorders in group 32), craniosynostosis syndromes (14 syndromes in group 33), dysostoses with predominant craniofacial involvement (19 disorders in group 34), dysostoses with predominant vertebral with and without costal involvement (eight syndromic or non-syndromic diseases in group 35), and four syndromes of patellar dysostoses (group 36). In total, 71 different genetic disorders have been associated with these diseases that show the premature fusion of cranial sutures or craniofacial, vertebral, costal, or patella ossification disorders. In zebrafish, 14 different genetic perturbations have been induced in the search for new models of disease: five for diseases in group 33, six for dysostoses in groups 34, one for group 35, and two for group 36 (Supplementary Table 1). Thirteen of these models are also considered by the international database ZFIN.

Mutations in the human *MSX2*, *TWIST1*, *TCF12*, *SKI*, and *MEGF8* genes are associated with Boston-type craniosynostosis, Saethre–Chotzen syndrome, coronal craniosynostosis, Shprintzen–Goldberg syndrome, and Carpenter syndrome, respectively (Mortier et al., 2019). The zebrafish orthologs *msx2* (Laue et al., 2011), *twist1* or *tcf12* (Teng et al., 2018), *ski* (Doyle et al., 2012), and *megf8* (Twigg et al., 2012) have been experimentally perturbed, and phenotypes were compared with symptoms of the above-mentioned diseases in group 33.

Chemical or TALEN mutants and morpholino downregulated embryos have been studied to analyze the diseases in zebrafish (Supplementary Table 1). Abnormalities of the cartilage during endochondral bone development, coronal craniosynostosis, or osteocyte differentiation of sutural cells are phenotypes that are observed after perturbation of zebrafish *msx2* gene (Laue et al., 2011). These phenotypes resemble symptoms in humans with Boston-type craniosynostosis. Moreover, the facial defects and lethality of zebrafish *twist1a/b* mutants, the loss of coronal sutures, and the abnormal growth of several head bones in *twist/tcf12* mutants mimic Saethre–Chotzen syndrome or coronal craniosynostosis symptoms (Teng et al., 2018). Furthermore, the maxillary hypoplasia, malformed ethmoid plate, micrognathia, microcephaly, ocular hypertelorism, spinal malformations, and severe cardiac defects shown by *skia/b* zebrafish morphants (Doyle et al., 2012) also resemble symptoms of Shprintzen–Goldberg syndrome. Finally, the heart-looping and epiboly defects, as well as other skeletal and left-right patterning abnormalities observed in *megf8* morphants (Twigg et al., 2012), mimic the symptoms of Carpenter syndrome in humans. These mutants and morphants provide good models of disease to researchers of group 33 disorders.

Various zebrafish models have been proposed for dysostoses with predominant craniofacial involvement. Models have been obtained for mutations in four human genes that cause mandibulofacial dysostosis (MFD). *TCOF1*, *POLR1C*, and *POLR1D* mutations are caused by Treacher Collins or Franceschetti–Klein syndrome, and mutations in *EFTUD2* gene cause *MFD* with microcephaly. Models of these diseases have been obtained after perturbation of zebrafish gene orthologs. These are *tcof1* (Weiner et al., 2012; Terrazas et al., 2017; Gil Rosas et al., 2019), *polr1c* (Lau et al., 2016; Noack Watt et al., 2016; Kwong et al., 2018), *polr1d* (Noack Watt et al., 2016), and *eftud2* (Lei et al., 2017; Wu J. et al., 2019). Mutations in human *POLR1A* gene that cause the Cincinnati type of acrofacial dysostosis and in *OFD1* gene that induce the orofaciodigital syndrome type I have also been modified to obtain models of diseases in group 34 (Mortier et al., 2019, Supplementary Table 1). Following mutations or downregulations of *polr1a* (Weaver et al., 2015; Watt et al., 2018) and *ofd1* (Ferrante et al., 2009) genes, phenotypes resembling human symptoms are obtained. These phenotypes are lethality, craniofacial disorders, small eyes, otic vesicles, bent pectoral fins, small body sizes, and abnormal hearts or notochords. These models have also been considered by ZFIN.

Mutant *choker* of zebrafish *meox1* gene (Dauer et al., 2018) has also been proposed as a Klippel–Feil syndrome model in group 35 (Supplementary Table 1), a disease caused by mutations in human ortholog *MEOX1* gene (see Mortier et al., 2019). The vertebral fusion, congenital scoliosis, and pectoral girdle asymmetry observed in this mutant almost exactly resembles the cervical vertebra fusions, congenital scoliosis, and skeletal alterations found in patients with this disease (Dauer et al., 2018).

Mutations in the human *LMX1B* and *CDC6* genes are associated with nail–patella syndrome and ear–patella–short stature syndrome (Meier–Gorlin syndrome), respectively (Mortier et al., 2019). Zebrafish orthologs *lmx1b* (Burghardt et al., 2013; Wang et al., 2019) and *cdc6* (Yao et al., 2017) have also been experimentally perturbed, and phenotypes were compared with these patellar dysostoses.

The translation of *lmx1bb* has been knocked down by specific morpholinos in zebrafish embryos. Morphants showed body curvature, coiled tail, severe cardiac abnormalities, body edema, pronephric defects, or eye development delay. These phenotypes were rescued by the injection of its mRNA (Burghardt et al., 2013; Wang et al., 2019). These phenotypes clearly resemble the symptoms of patients with nail–patella syndrome. ENU and CRISPR/Cas9 LOF and hypomorph mutants were obtained to study *cdc6* gene function (Yao et al., 2017). While the LOF leads to embryonic lethality by cell cycle arrest and an increase in apoptosis, hypomorphs are males with reduced body size, growth retardation, and defective reproduction (Supplementary Table 1). Besides phenotypic validation of this model, the authors also performed genetic analysis of human mutant alleles through overexpression of plasmid injections in embryos, adding a mechanistic knowledge to Meier–Gorlin syndrome (Yao et al., 2017).

### Models for Genetic Disorders Involving Aberrations in Fingers or Other Limb Elements Including Joints

The final six groups in the NCGSD, groups 37–42, include 105 human genetic diseases that produce skeletal disorders in fingers (brachydactyly, ectrodactyly, polydactyly, syndactyly, or triphalangism), limbs, or joints (Mortier et al., 2019). These disorders are arranged into brachydactyly with (10 diseases in group 38) or without (nine in group 37) extraskeletal manifestations; limb hypoplasia–reduction defects (28 diseases, most of them syndromic, in group 39); ectrodactyly, with and without other manifestations (10 disorders in group 40); a group including polydactyly, syndactyly, and triphalangisms (27 diseases in group 41); and a final group 42, which comprises four diseases with defects in joint formation and synostoses. These diseases are caused by 105 genetic disorders that show abnormalities of the limbs and face, finger or toe abnormalities, or internal organ defects such as kidney cysts, among others. These phenotypes have been associated with primary cilia (de Lange or Roberts syndrome) or cohesin complex (Meckel syndrome) defects. Only 15 zebrafish genes have been experimentally perturbed to model diseases in these groups, two for diseases in group 38, seven for diseases in group 39, and six for diseases in group 41 (Supplementary Table 1). These models are also considered by specialists in international repositories (ZFIN or EZRC), but there are other studies that suggest new incorporations to this set of models in a near future.

Mutations in the human *EP300* and *CHSY1* genes are associated with Rubinstein–Taybi syndrome and brachydactyly Temtamy type, respectively (Mortier et al., 2019). The zebrafish orthologs *ep300a/b* (Babu et al., 2018) and *chsy1* (Li et al., 2010) have been experimentally perturbed, and phenotypes compared with symptoms of these two brachydactylies in group 38. After knockdown of the *ep300a* and *ep300b* genes by morpholinos, jaw development defects with small heads and eyes, reduced pectoral fins, absence of the swim bladder, and pericardial edema were obtained. These phenotypes were generated by perturbation of skeletal progenitor cells (Babu et al., 2018) and resembled the symptoms of patients with Rubinstein–Taybi syndrome (Babu et al., 2018). Gene knockdown by morpholino injections and GOF by plasmid injection of zebrafish embryos have also been used to understand *chsy1* gene function during embryogenesis (Li et al., 2010). Similar phenotypes were found in both LOF and GOF studies, namely, reduced body size and pectoral fins, or malformations in the notochord, neurocranial cartilage, and inner ear. LOF studies also showed reduced eye distance and coloboma. The similarity shown by these phenotypes and the symptoms described in patients with brachydactyly Temtamy type (Li et al., 2010) strongly supports these models.

Mutations in 47 human genes cause reduction defects or hypoplasia in limbs in group 39 of the NCGSD (Mortier et al., 2019). These diseases vary in severity through a wide range of symptoms from missing fingers or toes to the complete absence of arms or legs. Experimental phenotypes with zebrafish were compared with symptoms shown by patients with these disorders and models for Cornelia de Lange syndrome, Fanconi anemia, Holt–Oram syndrome, or Roberts syndrome were proposed. *nipbl* (Muto et al., 2011; Pistocchi et al., 2013; Xu et al., 2015; Kawauchi et al., 2016), *smc1a* (Cukrov et al., 2018), *smc3*, and *rad21* (Xu et al., 2015) genes have been mutated or downregulated by morpholino injections to model de Lange syndrome. Of the 18 different causative genes, only *rad51* has also been mutated to model Fanconi anemia in zebrafish (Botthof et al., 2017). Mutations and morpholino knockdown of *tbx5a/b* genes have also been obtained to model Holt–Oram syndrome (Garrity et al., 2002; Parrie et al., 2013; Chiavacci et al., 2015; D′Aurizio et al., 2016). Finally, *esco2* gene has been downregulated by morpholino and retroviral insertions to model Roberts syndrome (Mönnich et al., 2011; Xu et al., 2013; Percival et al., 2015; Banerji et al., 2016; Supplementary Table 1). Some of these models have already been used for drug screening (see below).

Some of these syndromes are classified as cohesinopathies and partially share common phenotypes. Small head and eyes, cardiac edema, shortened body length, and curved tail were recurrent after the perturbation of genetic orthologs to those associated with de Lange syndrome (Muto et al., 2011; Pistocchi et al., 2013; Xu et al., 2015; Kawauchi et al., 2016; Cukrov et al., 2018). Moreover, craniofacial and pigmentation abnormalities, fin truncations, and regeneration defects associated with mitotic and apoptosis abnormalities were found when modeling Roberts syndrome after perturbation of the *esco2* ortholog (Supplementary Table 1). This group also includes disorders with complex and multisystemic symptoms, potentially useful for further genetic analysis and therapeutic research. For example, phenotypes in the kidney and hematopoietic stem and progenitor cells were found in the model for Fanconi anemia (Botthof et al., 2017), whereas fin and cardiac defects with looping failure have been described in the model for Holt–Oram syndrome (Garrity et al., 2002; Parrie et al., 2013; Chiavacci et al., 2015; D′Aurizio et al., 2016).

Finally, zebrafish models have also been proposed for disorders in the polydactyly–syndactyly–triphalangism group 41 (Supplementary Table 1). This is a group of diseases that shows congenital deformation of the upper extremity affecting digit morphology. Mutations in human genes have been associated with Cenani–Lenz syndactyly (*LRP4*) and Meckel syndrome types 1–5 (*MKS1*, *TMEM67*, *TMEM67*, *CEP290*, and *RPGRIP1L*) (Mortier et al., 2019) for which several zebrafish models have been generated. These models perturb the function of the genes *lrp4* (Tian et al., 2019), *mks1* (Leitch et al., 2008), *tmem216* (Valente et al., 2010), *tmem67* (Adams et al., 2012; Leightner et al., 2013), *cep290* (Leitch et al., 2008), and *rpgrip1l* (Khanna et al., 2009). Perturbations were obtained by ENU mutations or morpholino knockdown and phenotypes compared with disease symptoms.

The morpholino knockdown of *lrp4* leads to cyst formation at the fin and caudal plexus, malformed pectoral fin, and defective bones and kidney (Tian et al., 2019), phenotypes that resemble the symptoms of Cenani–Lenz syndactyly. The morpholino downregulation of *mks1*, *tmem216*, *tmem67*, *cep290*, or *rpgrip1l*, which code for transmembrane proteins or centrosome and cilium proteins, leads to gastrulation defects, short or curved body axis, or abnormal notochord and/or somites, phenotypes of ciliopathy defects (Supplementary Table 1). Disorders in other organs are renal cysts, otic vesicle anomalies, and hydrocephalus. These phenotypes resemble the symptoms of Meckel syndromes: enlarged kidneys, cysts, encephalocele, and dysplasia of bones (refs. in Supplementary Table 1).

## Complementary Studies

Additional zebrafish models have been proposed in which genes that interact with those causing diseases in the NCGSD are affected. For diseases in group 10, the morpholino knockdown of zebrafish *lpa1* and *atx* genes (Nishioka et al., 2016) has been proposed as a model for human dyschondroplasia. In this group, the CRISPR/Cas9 editing of *nomo* (Cao et al., 2018) or *msmo1* (Anderson et al., 2020) genes associated with deficiencies in the FGF signaling pathway or cholesterol biosynthesis has also been proposed as a model for chondrodysplasia. In this sense, perturbations in a protein involved in ribosome biogenesis and nucleologenesis, such as the one encoded by *pescadillo* gene, have been proposed as zebrafish models of metaphyseal dysplasia (Sun et al., 2019) or SBDS (Provost et al., 2012), diseases that are both in group 11.

The CRISPR/Cas9 editing of zebrafish *rpl13 gene* has been proposed for spondyloepimetaphyseal dysplasia (SEMD-RPL13 type), as recently found in several human families (Costantini et al., 2020). These additional zebrafish models further extend the offer of diseases affecting the epiphysis and metaphysis of long bones and vertebrae and could be key for understanding the mechanisms of disease governing genetic interactions of each of these disorders.

Our group has recently reported a new bent bone dysplasia (group 18) caused by mutations in *LAMA5*. Mutations in this laminin alter the focal adhesion pathway and the crosstalk of skeletal blood vessels and surrounding skeletal tissue (Barad et al., 2020). A mutation in zebrafish *lama5* gene was previously obtained, showing cell adhesion defects. This mutation showed phenotypes of skeletal disorganization in the fin fold and deformed skeletal elements of pectoral and caudal fins (Webb et al., 2007). This model could be useful to study this new bent bone dysplasia, to expand insights into the crosstalk between ECM and cell-to-cell adhesion signaling and to search for treatments.

Among the many examples, we finally focus on Potocki–Shaffer syndrome, caused by alterations of *PHF21A* gene, and osteochondrodysplasia, caused by mutations in *TAPT1B* gene. The downregulation of zebrafish *phf21a* by the injection of morpholino and mRNA rescue has supported a model for Potocki–Shaffer syndrome. Microcephaly, dysmorphism, autonomous spine and tail curvature, and pharyngeal cartilage defects (Kim et al., 2012) are some of the phenotypes shown by these morphants. Moreover, the morpholino downregulation and CRIPR/Cas9 editing of zebrafish *tapt1b* gene also support a model for osteochondrodysplasia. These morphants and mutants show craniofacial and pectoral fin defects, and delayed ossification that resemble the severe hypomineralization and multiple bone deformities shown by patients (Symoens et al., 2015). These two diseases could be included in group 29.

These are just some examples of the many studies found in the literature (i.e., Boyadjiev et al., 2006; Townley et al., 2008). Reviews can be found that introduce many of these alternative models (Luderman et al., 2017; Carnovali et al., 2019; Kwon et al., 2019; Busse et al., 2020; Tonelli et al., 2020a; Dietrich et al., 2021).

## Gaps and Future Perspectives

Many gaps in the screening of treatments against human diseases in zebrafish models have been previously published (Kwon et al., 2019). In this review, we will pay attention to a gap emerging from the potential pharmacological use of zebrafish models for NCGSD diseases. Although a huge variety of zebrafish models have been proposed, only a few have been used to evaluate treatments in scientific papers. In Supplementary Table 2, we show a significant group of 15 zebrafish models in which the testing of 30 potential treatments has been referenced. Diseases in groups 25 and 39 accumulate most of the drug testing (76.7%), with osteoporosis, OI, and de Lange syndrome being the most studied disorders. While genetic manipulations may be a future option, the chemical induction of osteoporotic human symptoms in wild-type zebrafish embryos is currently the most popular approach in these references (Supplementary Table 2). In this table, 12 of the 15 models are based on genetic modifications, but they have been used for only 13 of the 30 drugs tested.

In principle, chemical models used for drug testing have required prior biochemical and physiological analyses to obtain a deep comprehension of their suitability (Bae et al., 2017; Dietrich et al., 2021). In these cases, their current popularity may be grounded in this biochemical and cell biology comparison. This is undoubtedly the case of the osteoporosis assays (Dietrich et al., 2021), which may arise as a gold standard for the rest of models. Models in Supplementary Table 1 may also require further biochemical and/or cell biology comparison with human diseases to reach this standard.

On the contrary, genetic zebrafish models for other skeletal disorders are still awaiting to be proposed. This is supported by the number of disorder-causing genes in the NCGSD-2019 without zebrafish models. The skeletal expression of many zebrafish orthologs of these human genes has been studied by transcriptomic and *in situ* hybridization (references in ZIRC). This offers an important source of information for further genotype–phenotype associations and a better knowledge of skeletal homology and the conserved skeleton-related gene module. However, one of the most valuable sources of zebrafish for these comparative analyses is still to be exploited. An extensive list of unexplored mutants awaits in the zebrafish repositories, and a virtually unlimited list of knockdown manipulations with morpholinos or CRISPR/Cas9 editing are still to be performed. These new perturbations could also be useful for genetic analysis of proband genomes and deepening into the mechanisms of skeletal diseases. This lack of models and the still insufficient pool of patient samples for some of these diseases to demonstrate a link between gene mutations, mechanisms of disease, and symptoms are problems with pending solutions that require further research. In Supplementary Table 3, we show links to 438 sites on the ZIRC webpage that show mutant or transgenic lines with this pre-clinical potential.

Our review seeks to clarify the strength or limitations of each zebrafish model toward clinical applications. As strengths, our revision supports previous hypotheses of a conserved module of bone- (Kwon et al., 2019) or ciliopathy-related (Shim et al., 2016) genes in vertebrates. Moreover, after the review of zebrafish models for diseases in 27 NCGSD groups, we think that such hypotheses could be extended to OI and decreased bone density disorders (group 25), craniosynostosis (group 33), and dysostoses with predominant craniofacial involvement-related genes (group 34). Besides, we acknowledge the phenotypic similarity for the other less-studied 24 groups supporting the future applications of one or more conserved modules of skeletal-related developmental and homeostasis genes in vertebrates. As limitations, we identify models with significant phenotypic differences with humans (i.e., group 1, 17, 19, 27, or 31), which although may play a leading role in the study of skeletal evolutionary divergence within vertebrates, must be used with caution and acknowledging their differences.

In summary, the available zebrafish genetic models of disease, the use of some of these models for drug screening against skeletal diseases, and the ample availability of potential model mutants or transgenic lines at international repositories presumes a relevant offer of pre-clinical research for genetic skeletal disorders in humans. Due to this, new zebrafish models are arising at an exponential rate each year. This awakening interest in zebrafish models as a meeting point for basic and applied skeleton researchers goes far beyond pre-clinical drug testing and reveals a true “zebrafish revolution” as a low-cost alternative to rodents. A new thesis therefore needs to be considered.

## Data Collection Method

Articles revised in the review have been searched in PubMed, Google Scholar, ZFIN, and EZRC engines. The data in Supplementary Table 1 have been screened by reading articles searched in engines by key words related to “zebrafish” plus each name of disease or gene in NCGSD-2019. References in previous reviews were also searched and read. This general procedure has been performed twice. The main topics in these articles were annotated in an Excel datasheet. The data in Supplementary Table 2 have been obtained from articles searched in PubMed by key words related to “zebrafish,” the name of each disease, and the terms “drug,” “assay,” or “screening.” Specific terms such as glucocorticoids were also used. The articles selected for Supplementary Table 1 were also used after reading. The list of links in Supplementary Table 3 has been obtained by searching in the ZFIN and EZRC engines. These mutant or transgenic lines correspond to zebrafish gene ortholog to human genes in the NCGSD that are not present in Supplementary Tables 1, 2 and that have not been associated with zebrafish skeleton. This search was performed twice. An alternative search using “human” plus each name of disease in NCGSD was also performed. Articles published prior or contemporaneous to NCGSD-2019 were preferred.

## Author Contributions

MM-B and ID design the study and wrote the manuscript. AM-R performed data recollection. All authors contributed to the article and approved the submitted version.

## Conflict of Interest

The authors declare that the research was conducted in the absence of any commercial or financial relationships that could be construed as a potential conflict of interest.

## Publisher’s Note

All claims expressed in this article are solely those of the authors and do not necessarily represent those of their affiliated organizations, or those of the publisher, the editors and the reviewers. Any product that may be evaluated in this article, or claim that may be made by its manufacturer, is not guaranteed or endorsed by the publisher.
