# Supplementary material for: Zebrafish Models for Human Skeletal Disorders
Source: Front Genet. 2021 Aug 5;12:675331. doi: 10.3389/fgene.2021.675331 (PMC8418114; doi:10.3389/fgene.2021.675331)
Supplement: Supplementary file 1 [file Table_1.pdf]

Table 1. Models of human skeletal disorders in zebrafish

Group	Human Disease	Human Gene	Zebrafish models	Phenotype	References
**1.- FGFR3 chondrodysplasia group**	Thanatophoric dysplasia type 1<br>Thanatophoric dysplasia type 2	*FGFR3*	WT human and mutant zebrafish mRNA injection	Dorsalized embryos.	Lindy et al., 2016
**3.- Type 11 collagen group**	Stickler syndrome type 3 (non-ocular)	*COL11A2*	ENU mutant	Altered jaw and joint development. Impaired jaw function and premature adult osteoarthritis by craniofacial skeletal and chondrocyte abnormalities.	Lawrence et al.,2018
**9.- Ciliopathies with major skeletal involvement**	Short rib–polydactyly syndrome (SRPS) type 1/3 (Saldino–Noonan/Verma–Naumoff)	*IFT80*	Morfolino, RNA injection	Abnormal anterior neurocranium and curled tail. Pericardial edema. Short body size in double *ift80* + *bbs8 or bbs4* morphants.	Beales et al., 2007*; Hudak et al., 2010
	Asphyxiating thoracic dysplasia (ATD; Jeune)	*TCTEX1D2*	Morpholinos and/or RNA injection or retroviral insertion mutant	Reduced body size, ventrally curved body axis, craniofacial cartilage defects, kinked notochord, abnormal otoliths, or small eyes with retinal degeneration. Pericardial edema, endothelial autonomous intracranial hemorrhages, pronephric cysts and hydrocephalus.	Schmidts et al., 2014
		IFT80			Beales et al., 2007*; Hudak et al., 2010
		IFT172			Lunt et al., 2009; Halbritter et al., 2013; Bujakowska et al., 2015
		IFT81			Sun et al., 2004; Amsterdam et al., 2004; Dibella et al., 2009; Kallakuri et al., 2015; Dharmat et al., 2017; Eisa-Beygi et al., 2018; Petterson et al., 2018
		CEP120			Shaheen et al., 2015

	IFT140	Morfolino, RNA injection	Shortened body axis, deformed anterior structures and notochord, defects in somite size, tail extension defects.	Helm et al., 2017
	IFT52	ENU mutant, morpholino	Ventrally curved body axis, craniofacial cartilage defects, abnormal olfactory placode, pronephric cysts, and left-right asymmetry.	Zhao and Malicki, 2011; Dupont et al., 2019*
SRPS type 2 (Majewski)	*KIAA0753/si:dkey-243i1.1*	Morpholinos and/or RNA injection; ENU mutant	Ventrally curved body axis, craniofacial cartilage defects, abnormal otoliths, and small eyes. Pericardial edema, pronephric cysts and hydrocephalus.	Hammarsiö et al., 2017
	NEK1			Wheway et al., 2015
	IFT81	See IFT81, ATD (Jeune)	See IFT81, ATD (Jeune).	see IFT81, ATD (Jeune)
SRPS type 4 (Beemer)	*IFT122*	Morfolino, RNA injection	Shortened and ventrally curved body axis, cardiac edema, small eyes. Pronephric cysts, hydrocephalus, and otolith defects.	Walczak-Sztulpa et al., 2010
	IFT80	See SRPS type 1/3	See SRPS type 1/3.	Beales et al., 2007*; Hudak et al., 2010
Cranioectodermal dysplasia (Levin–Sensenbrenner) type 1, 2	*IFT122*	See SRPS type 4	See SRPS type 4.	Walczak-Sztulpa et al., 2010
	WDR19	Morpholinos (*wdr19/tect1)*	Convergence-extension defects, pronephric cysts, situs inversus, otolith defects. Hydrocephalus with *wdr19*MO.	Ryan et al., 2018
	IFT52	See ATD (Jeune)	See ATD (Jeune).	Zhao and Malicki, 2011; Dupont et al., 2019*
Mainzer–Saldino syndrome	*IFT140*	Morpholinos and/or RNA injection or retroviral insertion mutant	Gastrulation defects, ventral body-axis curvature, craniofacial cartilage defects, and abnormal otoliths. Situs inversus, pronephric cysts and hydrocephalus.	Helm et al., 2017
	IFT172			Lunt et al., 2009; Halbritter et al., 2013; Bujakowska et al., 2015

**11.- Metaphyseal dysplasias**	Cartilage-hair hypoplasia (CHH; metaphyseal dysplasia, McKusick type)	*RMRP*	CRISPR/Cas9 mutant	Wnt overexpression dependent chondrodysplasia. Ceratobranchial arches developmentally delayed or defective. Pharyngeal cartilage and teeth, ceratohyal cartilage, and basihyal cartilage defective. Ossification delayed in many head bones and enhanced in endochondral bones.	Sun et al., 2019
	Metaphyseal dysplasia with pancreatic insufficiency and cyclic neutropenia (Shwachman–Bodian–diamond syndrome, SBDS)	*SBDS*	Morpholino	Malformed cartilages in gill arches and lower jaw. Misshapen and thin inner ear bone. *p53*-dependent twisted tail.	Provost et al., 2012*
**13.- Spondylo-epi-(meta)-physeal dysplasias (SE(M)D)**	SEMD, Shohat type	*DDRGK1*	Morpholinos, mRNA injection, CRISPR/Cas9 (*rpl13*)	Craniofacial cartilage deformities at embryonic and juvenile stages.	Egunsola et al., 2017
	SEMD with immune deficiency, EXTL3 type	*EXTL3*	ENU mutant (*boxer)*	Defective thymopoiesis. Cartilage and pectoral fin defects.	Volpi et al., 2017
	SEMD with intellectual disability, NANS type	*NANS*	Morpholino (*nasna),* sialic rescue	Small head, pericardial edema, and craniofacial cartilage abnormalities.	van Karnebeek et al., 2016
	SPONASTRIME dysplasia	*TONSL*	CRISPR/Cas9 mutant	Lethal before 20 dpf, accelerated vertebra ossification, short body size and spinal abnormalities.	Burrage et al., 2019
**14.- Severe spondylodysplastic dysplasias**	Severe spondylometaphyseal dysplasia (SMD Sedaghatian-like)	*SBDS*	See SBDS (group 11)	See SBDS (group 11).	Provost et al., 2012*
**15.- Acromelic dysplasias**	Geleophysic dysplasia	*FBN1*	Morpholino	Expanded tail fin with vascular defects.	Chen et al., 2006
	Acromicric dysplasia	*FBN1*	See Geleophysic dysplasia	See Geleophysic dysplasia.	Chen et al., 2006
	Weill–Marchesani syndrome	*FBN1*			Chen et al., 2006
	Acrodysostosis	*PDE4D*	Morpholino, human mutant mRNA injection	Curved caudal fin, head increase, short and bent body. Pericardial edema.	Lindstrand et al., 2014

**17.- Mesomelic and rhizo-mesomelic dysplasias**	Dyschondrosteosis (Leri–Weill)	*SHOX*	Morpholinos	Growth retardation (reduced somite number and body length), reduced pectoral fins and decreased ossification in anterior vertebrae and craniofacial bones.	Kenyon et al., 2011; Sawada et al., 2015; Marchini et al., 2016; Montalbano et al., 2016
	Mesomelic dysplasia, Langer type	*SHOX*	See Dyschondrosteosis (Leri–Weill).	See Dyschondrosteosis (Leri–Weill).	Marchini et al., 2016; Montalbano et al., 2016
	Robinow syndrome, dominant type	*WNT5A*	Human WT, DN and hypomorph mRNA injections	Insulin-expressing cell coalescence in pancreatic islet, and axis duplication. *pipetail*-like bent tail, not in DN.	Person et al., 2010
**18.- Bent bone dysplasia group**	Campomelic dysplasia (CD)	*SOX9*	ENU mutant *(jellyfish, sox9a).* eGFP or mCherry assays	Craniofacial, neurocranium pharyngeal arches, and pectoral girdle cartilage defects.	Yan et al., 2002; Nissen et al., 2006; Plavicki et al., 2014; Gordon et al., 2014
**19.- Primordial dwarfism and slender bones group**	Saul–Wilson syndrome	*COG4*	CRISPR/Cas9 mutant	Malformed inner ear and semicircular canals. Few hair bundles in hair cells and neuromasts. Reduced response to auditory stimuli. Homozygotes with shorter bodies, craniofacial defects, and stubby ‘clubbed’’ pectoral fins.	Ferreira et al., 2018*
**20.- Dysplasias with multiple joint dislocations**	Multiple joint dislocations with amelogenesis imperfecta	*SLC10A7*	Morpholino	Teeth cartilage bent downwards, edema in whole body, reduced head, and eyes. Curled body and craniofacial disorders.	Ashikov et al., 2018
	Severe (lethal) neonatal short limb dysplasia with multiple dislocations	*FAM20B*	ENU mutants (*b1125*/*b1127)* and cDNA injection	Primary chondrocyte and perichondral bone defects.	Eames et al., 2011
	Ehlers–Danlos syndrome, kyphoscoliotic type 1	*PLOD1*	Morpholino (*plod1a)*	Absent actinotrichia, ventral fin fold reduced or deformed, post-vent region curved dorsal.	Durán et al., 2011
**23.- Osteopetrosis and related disorders**	Osteopetrosis, severe neonatal or infantile forms	*CLCN7*	Morpholino	Craniofacial cartilage defects and dental malformations.	Zhang et al., 2019
	Osteopetrosis, intermediate form	*CLCN7*			
	Osteopetrosis, late-onset form type 2 (OPTA2)	*CLCN7*			

	Dysosteosclerosis	*CSF1R*	ENU (*csf1ra*), TALEN (*csf1rb)* mutants	Small vertebral arches and brain microglia deficiency.	Oosterhof et al., 2019
**24.- Other sclerosing bone disorders**	Hyperostosis–Hyperphosphatemia syndrome	*GALNT3*	TALEN and ENU mutants.	Hyperostosis and ectopic calcium deposits.	Stevenson et al., 2017*; Bergen et al., 2017
	Lenz–Majewski hyperostotic dysplasia	*PTDSS1*	Stable ubiquitous or cell-specific expression of WT and mutant human forms by transpose-mediated transgenesis	Mild vertebrae scoliosis with incomplete penetrance.	Seda et al., 2019
**25.- Osteogenesis Imperfecta and decreased bone density group**	Classic osteogenesis imperfecta types 1-4 (with mutations in collagen genes)	*COL1A1*	ENU mutant (*med*). Several mutants	Small and malformed cartilages and severe craniofacial dysmorphology, delayed bone ossification, undulation of the larval fin, and ray fractures not repaired. Bone fragility.	Asharani et al., 2012; Fiedler et al., 2018; Gistelinck et al., 2018
		COL1A1	ENU mutant (*Chi*)	Defective bone growth and density.	Fisher et al., 2003; Gioia et al., 2017; Fiedler et al., 2018; Gistelinck et al., 2018; Enderli et al., 2016
		COL1A1	ENU mutants	Defective bone density.	Fiedler et al., 2018; Gistelinck et al., 2018
		COL1A2	Quantitative ENU mutant	Kyphosis, slightly lower mineralized and reduced in thickness bones, half thickness dermis. Misshapen and over-mineralized axial and cranial skeleton.	Gistelinck et al., 2018
	Osteogenesis imperfecta types with mutations in non-collagen genes	*CRTAP*	CRISPR/Cas9 mutant	Growth delay, short size, head and body disproportion, chunky head, deformed spine with vertebral fusions and compressions, calli in ribs, delayed bone mineralization, swim bladder inflation, and high mortality.	Tonelli et al., 2020b*
		LEPRE1	CRISPR/Cas9 (*p3h1)* mutant	Growth delay, short size, head and body disproportion, deformed spine with vertebral fusions and compressions, calli in ribs, delayed bone mineralization and swim bladder inflation.	

		BMP1	ENU mutant (*frilly fins*/sa2416, *bmp1a)*. WT and mutant human mRNA injection	Short body axis and malformed craniofacial bones, vertebra, and fin. Reduced vertebra ossification and bone density with defective repair of fractures. Over-mineralization.	Asharani et al., 2012; Cho et al., 2014; Gistelinck et al., 2018; Hur et al., 2017*, Tomecka et al., 2019; rev. Enderli et al., 2016
		SPARC	Morpholino, mRNA injection	Deformed otoliths, pharyngeal cartilages, and inner ear.	Rotllant et al., 2008; Kang et al., 2008
	Osteoporosis—X-linked form	*PLS3*	Morpholino, human mRNA injection	Craniofacial dysplasia. Impaired muscles.	van Dijk et al., 2013: rev. Besio et al., 2019
		MBTPS2	Morpholino, ENU mutant (*mbtps1)*	Pharyngeal arch 3-7 chondrocyte disorganized, abnormal.	Schlombs et al., 2003; rev. Besio et al., 2019
	Bruck syndrome type 2 (BS2)	*PLOD2*	ENU mutant	Short body axis, severe skeletal abnormalities. Bone fragility and fracture. Scoliotic spine, compressed and distorted vertebrae, excessive bone formation at vertebral ends, high mineral density in vertebral centra, and musculoskeletal abnormalities.	Gistelinck et al., 2016, 2018; Hur et al., 2017*
	Cole–Carpenter like dysplasia	*SEC24D*	ENU mutant (*bulldog)*, morpholino	Severe craniofacial dysmorphology with small and malformed cartilages. Collagen type II secretion failure and ER stress.	Sarmah et al., 2010; Garbes et al., 2015
	Cutis laxa, autosomal recessive form, type 2B (ARCL2B)	*PYCR1*	TALEN mutant	Aging phenotypes: dwarfism, slow swimming, fertility loss at six months, increased senescence, and mortality.	Liang et al., 2019*
	Short stature, optic nerve atrophy and Pelger-Huet anomaly (SOPH syndrome)	*NBAS*	Morpholino	Chondrocranial defects, growth retardation, bent body axis, curvy tail.	Palagano et al., 2018
**26.- Abnormal mineralization group**	Vitamin D-dependent rickets, type 1B	*CYP2R1*	Morpholino (*vdra/b)*	Impaired vertebra ossification.	Lin et al., 2012; Kwon, 2019
	Vitamin D-dependent rickets, type 2A	*VDR*			

**27.- Lysosomal storage diseases with skeletal involvement (dysostosis multiplex group)**	Mucopolysaccharidosis type 2	*IDS*	Morpholino, enzymatic rescue. CRISPR/Cas9 mutant	Misshapen trunk and facial cartilage formation. Altered migration of neural crest cells, spine kyphosis and scoliosis, and hepatomegaly.	Moro et al., 2010; Bellesso et al., 2018*
	Mucolipidosis II (I-cell disease), alpha/beta type	*GNPTAB*	Morpholino, TALEN mutant	Short size, absence of pectoral fins, pericardial edema, enlarged hearts, small misshapen eyes, blunt, rounded neurocraniums, and abnormal otoliths, and otic vesicles. Motility defect, lack swim bladders, and reduced yolk consumption.	Flanagan-Steet et al., 2009*; Petrey et al., 2012*; Flanagan-Steet et al., 2016*; 2018*
**28.- Osteolysis group**	Mandibuloacral dysplasia	*LMNA*	Morpholino, Transgenic overexpression of mutant human alleles	Skin, muscle, adipose tissue, and craniofacial abnormalities/cartilage defects associated to apoptosis and cell-cycle arrest.	Koshimizu et al., 2011
	Multicentric osteolysis, nodulosis and arthropathy (MONA)	*MMP14*	CRISPR/Cas9 mutant	Craniofacial malformations, kyphosis, short-stature, reduced bone density, and collagen remodeling defects.	de Vos et al., 2018
**29.- Disorganized development of skeletal components group**	Multiple cartilaginous exostoses (osteochondromas)	*EXT2*	ENU mutant (*dackel*) and FGF8 bead implants	Cartilage condensation, hypertrophic chondrocytes, and ossification defects. Teeth have abnormal morphology.	Clément et al., 2008; Wiweger et al., 2012*, 2014*
	Fibrodysplasia ossificans progressiva (FOP)	*ACVR1/acvr1l*	Mutant, mutant mRNA injection, GOF *mCherry*-tagged, heat-shock-inducible transgenic lines	Loss of ventral tail tissue (LOF). Heterotopic ossification lesions, spinal lordosis, vertebral fusions, and malformed pelvic fins (GOF).	Shen et al., 2009; LaBonty et al., 2017*, LaBonty and Yelick, 2019*; Mucha et al., 2018*
**30.- Overgrowth (tall stature) syndromes with skeletal involvement**	Marfan syndrome	*FBN1*	Morpholino	Expanded ventral fin fold, situs inversus and pronephros cysts.	Chen et al., 2006
**31.- Genetic inflammatory/rheumatoid-like osteoarthropathies**	Progressive pseudorheumatoid dysplasia (PPRD; SED with progressive arthropathy)	*WISP3/ccn6*	Morpholinos, mRNA injection	Dorsoventral patterning affected (OE). Mandibular and pharyngeal cartilage size and shape affected (KO).	Nakamura et al., 2007

**33.- Craniosynostosis syndromes**	Craniosynostosis Boston type	*MSX2*	ENU amorph and hypomorph mutants	Amorph shows fusions or overgrowth of cartilages during endochondral bone development. Hypomorph causes coronal craniosynostosis by accelerated gaining of osteocyte features by sutural cells.	Laue et al., 2011*
	Saethre–Chotzen syndrome	*TWIST1*	TALEN (Single *twist1a, twist1b, tcf12*; double *twist1a; twist1b*, *twist1b; tcf12)* mutants	Facial defects and lethality of double *twist1a/b* mutant partially rescued with *tcf12* mutation. Loss of coronal suture, and abnormal growth of calvaria, front and parietal bones in double *twist/tcf12* mutant.	Teng et al., 2018*
	Coronal craniosynostosis	*TCF12*			
	Shprintzen–Goldberg syndrome	*SKI*	Morpholino (*skia/b)*	Maxillary hypoplasia, malformed ethmoid plate, micrognathia, microcephaly, frequent ocular hypertelorism and spinal malformations. Severe cardiac defects.	Doyle et al., 2012*
	Carpenter syndrome	*MEGF8*	Morpholino, RNA injection	Heart-looping and epiboly defects, short wide notochord, and abnormal left-right patterning.	Twigg et al., 2012*
**34.- Dysostoses with predominant craniofacial involvement**	Mandibulofacial dysostosis (Treacher Collins, Franceschetti–Klein)	*TCOF1*	Morpholino, ENU mutant	Craniofacial cartilage disorders by increased cell death during CNCC development.	Weiner et al., 2012*; Terrazas et al., 2017: Gil Rosas et al., 2019*
		POLR1C	ENU mutant, morpholino	Craniofacial cartilage disorders by increased cell death during CNCC development.	Lau et al., 2016*; Noack Watt et al., 2016*; Kwong et al., 2017*
		POLR1D	ENU mutant	Craniofacial cartilage disorders by increased cell death during CNCC development.	Noack Watt et al., 2016*
	Mandibulofacial dysostosis with microcephaly	*EFTUD2*	TALEN mutant, morpholinos	Lethality. Mandibular, ceratobranchial cartilage, notochord, and otoliths defects.	Lei et al., 2017*; Wu et al., 2019b*

	Acrofacial dysostosis, Cincinnati type	*POLR1A*	ENU mutant	Smaller eyes, otic vesicles, pectoral fins, and body size. Abnormal heart and otoliths. Craniofacial phenotype by reduced formation of NCC-derived elements. Pericardial edema. Swim bladder inflation failure. Lethality.	Watt et al., 2018*; Weaver et al., 2015*
	Orofaciodigital syndrome type I (OFD1)	*OFD1*	Morpholino	Ciliopathy phenotypes: bent body axes, hydrocephalus, edema, and convergent-extension defects.	Ferrante al., 2008; rev. Varga et al., 2018
**35.- Dysostoses with predominant vertebral with and without costal involvement**	Klippel-Feil syndrome	*MEOX1*	ENU mutant (*choker)*	Vertebral fusion, congenital scoliosis, and pectoral girdle asymmetry.	Dauer et al., 2018*
**36.- Patellar dysostoses**	Nail-patella syndrome	*LMX1B*	Morpholino (*lmx1bb)*, mRNA injection	Body curvature, coiled tail, severe cardiac, body edema, pronephric or eye developmental defects.	McMahon et al., 2009; Burghardt et al., 2013*; Wang et al., 2019*
	Ear-patella-short stature syndrome (Meier–Gorlin)	*CDC6*	ENU and CRISPR/Cas9 amorph and hypomorph mutants. Plasmid injection with human mutant forms	Embryonic lethal by cell cycle arrest and extensive apoptosis (LOF). Males with reduced body size, growth retardation and defective reproduction (Hypomorphs).	Yao et al., 2017*
**38.- Brachydactylies (with extraskeletal manifestations)**	Rubinstein–Taybi syndrome	*EP300*	Morpholino (*ep300a*/*b)*	Jaw development defects, small heads and eyes and absence of swim bladder. Short jaw, reduced pectoral fins and pericardial edema by progenitor cell disorders.	Babu et al., 2018*
	Brachydactyly, Temtamy type	*CHSY1*	Morpholino (LOF), plasmid injection (GOF)	Reduced body length and pectoral fins. Notochord, neurocranial cartilage and inner ear disorders (LOF, GOF). Reduced eye distance and colobomas (LOF).	Li et al., 2010*
**39.- Limb hypoplasia–reduction defects group**	de Lange syndrome	*NIPBL*	Morpholino (*nipbla/b)* and LiCl	Small head and eyes, cardiac edema, short body length and curved tail. Defects in heart, circulation, and size and patterning of pectoral fin and gut. Larval lethality.	Pistocchi et al., 2013*, Xu et al., 2015*; Kawauchi et al., 2016*; Muto et al., 2011*

		SMC1A	ENU mutant	Small eyes, cardiac edema, and loss of circulation. Lethality.	Cukrov et al., 2018*
		SMC3	Morpholino	Small head and eyes, cardiac edema, short body length and curved tail.	Xu et al., 2015*
		RAD21	Morpholino	Small head and eyes, cardiac edema, short body length and curved tail.	Xu et al., 2015*
	Fanconi anemia (see note below)	*RAD51, FANC, BRCA2, FANCD2, FANCE, FANCF, FANCG, FANCI, FANCJ, FANCL, FANCN, FANCO, FANCP, FANCQ, FANCR, FANCT, FANCU, FANCV*	ENU mutant (*rad51)*	Hypocellular kidney marrow, sensitivity to cross-linking agents, decreased body size and microphthalmia. Decreased proliferation and increased apoptosis of embryonic hematopoietic stem and progenitor cells.	Botthof et al., 2017*
	Holt–Oram syndrome	*TBX5*	ENU mutant (*heartstrings*), morpholinos (*tbx5a/b)*	Fin and cardiac defects with looping failure.	Garrity et al., 2002*; Chiavacci et al., 2015*; Parrie et al., 2013*; D'Aurizio et al., 2016*
	Roberts syndrome	*ESCO2*	Morpholino, retroviral insertion mutant	Mitotic and apoptosis defects, craniofacial and pigmentation abnormalities, fin truncations, and regeneration defects.	Mönnich et al., 2011*; Xu et al., 2013*; Percival et al., 2015*; Banerji et al., 2016*
**41.- Polydactyly-Syndactyly-Triphalangism group**	Cenani–Lenz syndactyly	*LRP4*	ENU mutant, morpholino	Cyst formation at fin and caudal vein plexus, malformed pectoral fin, defective bone, and kidney formation.	Tian et al., 2019*
	Meckel syndrome type 1	*MKS1*	Morpholino, WT and mutant human mRNA injection	Gastrulation defective.	Leitch et al., 2008; rev. Varga et al., 2018
	Meckel syndrome type 2	*TMEM216*	Morpholino	Gastrulation defective. Short body and axis. Broad notochord and misshapen somites.	Valente et al., 2010

Meckel syndrome type 3	*TMEM67*	Morpholino	Ciliopathy defects: dorsal body axis curvature, renal cysts, variable otic vesicle anomalies, notochord defects, and hydrocephalus.	Adams et al., 2012*; Leightner et al., 2013*
Meckel syndrome type 4	*CEP290*	Simple and double (*msk3)* morpholino	Gastrulation defective. Short body axes, wide and thin somites, and broad and kinked notochord.	Leitch et al., 2008; rev. Varga et al., 2018
Meckel syndrome type 5	*RPGRIP1L*	Morpholino, WT and mutant human mRNA injection	Gastrulation defects: short body axis, thin somites with broad lateral extensions, kinked notochord, underdeveloped anterior structures, and tail extension abnormalities. Retinal degeneration.	Khanna et al., 2009; rev. Varga et al., 2018

Note: Diseases order according to Mortier et al., 2019. Name of genes in zebrafish models column occur if paralogs or other genes are also studied. The nature of mutation is stated when necessary. *models of disease referenced at ZFIN.
