# Supplementary material for: Zebrafish Models for Human Skeletal Disorders
Source: Front Genet. 2021 Aug 5;12:675331. doi: 10.3389/fgene.2021.675331 (PMC8418114; doi:10.3389/fgene.2021.675331)
Supplement: Supplementary file 2 [file Table_2.pdf]

Table 2. Drugs tested in zebrafish models of skeletal disorders

Nosology group	Human disease	Zebrafish model	Drug	Reference
**8**	Spondylocarpotarsal synostosis syndrome	TALEN *smyhc* mutant	para-aminoblebbistatin	Whittle et al., 2020
**11**	Cartilage-hair hypoplasia	CRISPR-Cas9 *rmrp* mutant	XAV939	Sun et al., 2019
**23**	Osteopetrosis/pycnodysostosis	*clcn7* morphant	SB431542	Zhang et al., 2019
**25**	Osteogenesis imperfecta	Chihuahua mutant	4-PBA	Gioia et al., 2017
	Osteoporosis	Callus repair in *bmp1a* and *csf1ra* mutants	Alendronate sodium trihydrate	Tomecka et al., 2019
		Dexamethasone-induced Osteoporosis	Tanshinol	Luo et al., 2015
			Tetrahydroxystilbene glucoside	Zheng et al., 2017
			Zuogui Pill	Yin et al., 2018
		Prednisolone-induced Osteoporosis	Salvianolic acid B	Luo et al., 2016
			Baicalin	Zhao et al., 2020
			Alendronate	Pasqualetti et al., 2015
			anandamide and N-linoleoylethanolamine	Carnovali et al., 2016
			Xian-ling-gu-bao (XLGB)	Wu et al., 2017
			Citrofulvicin	Chen et al., 2018
			ABPB-3	Zhang et al., 2018
			Nacreous protein N16	Lin et al., 2019
			Acaulide	Wang et al., 2018a
			Acaulins A and B,	Wang et al., 2018b
			Lentinula ethyl acetate fraction	Lee et al., 2020
		Prednisone-induced Osteoporosis	Sanggenon C	Wang et al., 2018c
**34**	Treacher-Collins syndrome	*tcof1*-MO injection	Proteosomes inhibitors MG132 and Bortezomib	Gil Rosas et al., 2019
**38**	Rubinstein Taybi Syndrome	*ep300* knockdown	HDACi III, CHIC35	Babu et al., 2018
**39**	Cornelia de Lange syndrome	SMC1a mutant	Iboceine	Cukrov et al., 2018
		nipblb morphants	lithium chloride	Pistocchi et al., 2013
		nipbla/b, *rad21* and *smc3*-morphants	L-leucine, alpha-ketoiso-caproate	Xu et al., 2015
	Roberts syndrome	*esco2* mutants and morphants	L-leucine	Xu et al., 2013
