# Supplementary material for: Zebrafish Models for Human Skeletal Disorders
Source: Front Genet. 2021 Aug 5;12:675331. doi: 10.3389/fgene.2021.675331 (PMC8418114; doi:10.3389/fgene.2021.675331)
Supplement: Supplementary file 3 [file Table_3.pdf]

Table 3. Examples of mutant/transgenic strains candidate for zebrafish models of an NCGSD disease

Group	Disease	Human Gene	ZF ortholog	Mutant/Transgenic strains	
**4. Sulphation disorders**	Chondrodysplasia gPAPP type (includes Catel–Manzke-like syndrome)	*IMPAD1*	*bpnt2*	sa31262	
**7. Filamin group and related disorders**	Frontometaphyseal dysplasia	FLNA	*flna*	sa17303	sa24296
				sa24297	sa24298
				sa32439	sa43949
				sa43950	sa43951
		MAP3K7	*map3k7*	sa230	sa23699
				la021478Tg	
		TAB2	*tab2*	sa6611	sa36936
				sa36937	la013779Tg
				la021394Tg	
**9. Ciliopathies with major skeletal involvement**	Chondroectodermal dysplasia (Ellis-van Creveld)/SRPS type 5/SRPS unclassified (Cranioectodermal dysplasia (Levin–Sensenbrenner) type 1, 2	*WDR35*	*wdr35*	sa14117	sa22338
				sa35532	sa42238
				w150*	
	Short rib–polydactyly syndrome (SRPS) type 1/3 (Saldino–Noonan/Verma–Naumoff)	*WDR60*	*wdr60 (dync2i1)*	sa34135	
	SRPS unclassified	*INTU*	*intu*	fh347	
		FUZ	*fuz*	fh345	fh346
				sa32831	fuz_unrecovered
**10. Multiple epiphyseal dysplasia and pseudoachondroplasia**	Multiple epiphyseal dysplasia (MED)	*COL9A1*	*col9a1a*	sa15187	sa27738
**11. Metaphyseal displasias**	Metaphyseal dysplasia with pancreatic insufficiency and cyclic neutropenia (Shwachman-Bodian-diamond syndrome, SBDS)	*EFL1*	*efl1*	sa12887	sa30883
				sa20868	sa34011
		DNAJC21	*dnajc21*	sa25150	sa37242

		SRP54	*srp54*	sa421	sa11820
				sa36339	
**12. Spondylometaphyseal dysplasias (SMD)**	Spondyloenchondrodysplasia (SPENCD)	*ACP5*	*acp5a*	la010828Tg	sa17411
				sa16094	sa40635
			acp5b	sa14177	sa16152
	SMD with cone-rod dystrophy	*PCYT1A*	*pcyt1ab*	sa9717	sa23376
				sa13515	
**13. Spondylo-epi-(meta)-physeal dysplasias (SE(M)D)**	Dyggve–Melchior–Clausen dysplasia (DMC)	*RAB33B*	*rab33ba*	ni104*	
	SED with diabetes mellitus, Wolcott–Rallison type	*EIF2AK3*	*eif2ak3*	it312*	sa451
				sa4496	sa42159
				la018397Tg	
	SEMD with leukodystrophy, AIFM1 type	*AIFM1*	*aifm1*	sa1710	sa33579
				sa40408	
	SEMD with intellectual disability, RSPRY1 type	*RSPRY1*	*rspry1*	sa16314	sa32199
				sa23276	sa43079
				sa23277	
	SEMD, PISD type	*PISD*	*pisd*	la025197Tg	
	SEMD, UFSP2 type	*UFSP2*	*ufsp2*	sa1746	zko339b
	SEMD, short limb–abnormal calcification type	*DDR2*	*ddr2a/b*	la015902Tg	sa19669
	SED tarda, X-linked (SED-XL)	*TRAPPC2*	*trappc2*	sa38768	
	Ehlers–Danlos syndrome, spondylodysplastic type	*SLC39A13*	*slc39a13*	sa31578	
	CODAS syndrome	*LONP1*	*lonp1*	sa39354	la022151Tg
				sa43743	
	CAGSSS syndrome	*IARS2*	*iars2*	sa2910	sa17843
				sa14246	sa28862
**14. Severe spondylodysplastic displasias**	Schneckenbecken dysplasia	*SLC35D1*	*slc35d1b*	sa25098	
	Opsismodysplasia	*INPPL1*	*inppl1b*	sa22511	sa22512
	MAGMAS related skeletal dysplasia	*PAM16*	*pam16*	sa40010	sa44557
**15. Acromelic dysplasias**	Weill–Marchesani syndrome	*ADAMTS10*	*adamts10*	sa7130	sa31645
				sa9421	sa34374
				sa18917	sa38685
		LTBP2	*ltbp3*	sa20482	sa44612
				sa20483	la015669Tg
				sa40507	

**17. Mesomelic and rhizo-mesomelic dysplasias**	Mesomelic dysplasia, Savarirayan type (triangular tibia-fibular aplasia)	*ID4*	*id4*	bns18*	
**18. Bent bone dysplasia**	Stüve–Wiedemann dysplasia	*LIFR*	*lifra*	sa26400	
			lifrb	sa6671<br>sa29572	sa17514
**19. Primordial dwarfism and slender bones**	3-M syndrome	*OBSL1*	*obsl1a*	sa9215	sa25336
				sa11178	sa33862
				sa14871	sa33863
				sa20691	sa33864
				sa25335	
			obsl1b	sa7206	sa34721
				sa10142	sa41505
				sa15355	
	Sanjad–Sakati syndrome	*TBCE*	*tbce*	la012057Tg	
	MOPD2 Majewski type	*PCNT2*	*pcnt*	sa7031<br>sa33823<br>tup5*	la029171Tg<br>tup2*
	Microcephalic osteodysplastic primordial dwarfism (other types)	*CEP152*	*cep152*	sa13346	sa24712
				sa38112	
		DNA2	*dna2*	sa773	sa35476
				sa8393	sa42185
				sa35475	
		TRAIP	*traip*	sa21949	sa44745
		CENPE	*cenpe*	sa19390	sa24832
				sa32570	sa32571
				zko13a	
		XRCC4	*xrcc4*	sa13686	sa34984
				sa34985	
	IMAGE syndrome	*POLE*	*pole*	sa6963	sa10893
				sa26416	sa31416
				sa44601	t20320*
				la024788Tg	
	Desbuquois dysplasia type 1/Kim type/MED recessive type	*CANT1*	*cant1a*	la015280Tg	
			cant1b	sa35167	
	SEMD-JL Beighton type	*B3GALT6*	*b3galt6*	sa41758	
**20. Dysplasias with multiple joint dislocations**	SEMD with joint laxity (SEMD-JL), EXOC6B type	*EXOC6B*	*exoc6b*	sa20933	sa40885
				sa34065	sa40886
				sa34066	la029306Tg

	Short stature with joint laxity and myopia	*GZF1*	*gzf1*	sa9486	sa29449
**21. Chondrodysplasia punctata (CDP)**	CHILD	*NSDHL*	*nsdhl*	sa35670	sa35671
	Rhizomelic CDP	*PEX7*	*pex7*	sa32312	
		AGPS	*agps*	sa13105	sa34543
				sa14227	sa38719
				sa21415	sa41334
				sa34542	
		FAR1	*far1*	sa13327	sa39476
		PEX5	*pex5*	sa10186	
**22. Neonatal osteosclerotic dysplasias**	Desmosterolosis	*DHCR24*	*dhcr24*	sa3089	sa5668
				sa15847	la013955Tg
				la021775Tg	
	Raine dysplasia	*FAM20C*	*fam20ca*	sa11878	sa38414
**23. Osteopetrosis and related disorders**	Osteopetrosis, severe neonatal or infantile forms	*TCIRG1*	*tcirg1a*	sa1773	la021218Tg
	Osteopetrosis, OPTB5	*OSTM1*	*ostm1*	sa14055	sa37077
	Osteopetrosis, intermediate form	*TNFSF11*	*tnfsf11*	sa38731	
		PLEKHM1	*plekhm1*	sa6231	
	Osteopetrosis, LAD3	*FERMT3*	*fermt3a*	sa11472	sa31488
				sa31489	sa33766
				sa40594	sa45237
			fermt3b	sa17885	
	Osteoclerotic metapkyseal dysplasia	*LRRK1*	*lrrk1*	sa12644	sa13011
				sa13125	sa13187
				sa30882	sa40838
**24. Other sclerosing bone disorders**	Osteopoikilosis/Melorheostosis with osteopoikilosis	*LEMD3*	*lemd3*	sa31386	sa40250
	Melorheostosis	*MAP2K1*	*map2k1*	sa6511	sa43086
	Craniometaphyseal dysplasia	*ANKH*	*ankha*	sa37832	
	Diaphyseal dysplasia Camurati–Engelmann	*TGFB1*	*tgfb1b*	sa3109	zf2126*
				ihb421	bns354*
	Hyperostosis–Hyperphosphatemia syndrome/Hypophosphatemic rickets, autosomal dominant (g.26)	*FGF23*	*fgf23*	sa18050	
	Hyperostosis–Hyperphosphatemia syndrome	*KL*	*kl*	sa12515	sa18644
				sa41699	sa41700
	Hypertrophic osteoarthropathy	*HPGD*	*hpgd*	sa7243	
	Diaphyseal medullary stenosis with malignant fibrous histiocytoma	*MTAP*	*mtap*	sa39619	
**25. Osteogenesis Imperfecta and decreased bone density**	OI type 3/OI type 4/ Osteoporosis—AD form	*WNT1*	*wnt1*	sa24353	tud49Tg*
				tud50Tg	

		CREB3L1	*creb3l1*	sa40963	
		TENT5A	*tent5aa*	sa16593	
	Cole–Carpenter dysplasia (bone fragility with craniosynostosis)	*P4HB*	*p4hb*	sa11106	sa26724
				sa31506	
	Spondylo-ocular dysplasia	*XYLT2*	*xylt2*	sa25267	
	Gnathodiaphyseal dysplasia	*ANO5*	*ano5a*	sa24725	sa38139
				sa25238	
			ano5b	sa40934	
	Geroderma osteodysplasticum	*GORAB*	*gorab*	sa14159	
	Wiedemann–Rautenstrauch syndrome	*POLR3A*	*polr3a*	sa907	sa31896
				sa35377	sa38910
				sa44776	
**26. Abnormal mineralization**	Hypophosphatemic rickets, autosomal recessive, type 1 (ARHR1)	*DMP1*	*dmtf1*	sa33393	sa40225
	Hypophosphatemic rickets with hypercalciuria, X-linked	*CLCN5*	*clcn5b*	sa20918	
	Hypophosphatemic rickets with hypercalciuria, autosomal recessive (HHRH)	*SLC34A3*	*slc34a2a*	sa12038	sa32626
			slc34a2b	sa37585	la022442Tg
	Vitamin D-dependent rickets, type 1B	*CYP2R1*	*cyp2r1**	ihb153*	sa7595
				zf734*	la029312Tg
				la029313Tg	ihb152*
**27. Lysosomal storage diseases with skeletal involvement (dysostosis multiplex group)**	Mucopolysaccharidosis type 1H-1S	*IDUA*	*idua*	sa14162	sa40364
				sa38450	la025333Tg
	Mucopolysaccharidosis type 3B	*NAGLU*	*naglu*	sa13573	
	Mucopolysaccharidosis type 3D	*GNS*	*gnsa*	sa17618	
	Mucopolysaccharidosis type 4A	*GALNS*	*galns*	sa21091	sa41036
				sa31601	sa41037
				sa31602	
	Mucopolysaccharidosis type 4B/ GM1 Gangliosidosis, several forms	*GLB1*	*glb1*	sa8423	sa11023
				sa18711	sa19602
				sa24857	
	Mucopolysaccharidosis-plus syndrome (VPS33A deficiency)	*VPS33A*	*vps33a*	sa18831	
	Fucosidosis	*FUCA*	*fuca1.1*	sa18262	sa44919

			fuca1.2	sa15377	sa29230
				sa18216	sa43309
				sa23558	
	Alpha-Mannosidosis	*MAN2B1*	*man2b1*	sa4425	sa31840
				sa7293	sa41874
				sa12135	
	Aspartylglucosaminuria	*AGA*	*aga*	sa35733	la027552Tg
	Sialic acid storage disease (SIASD)	*SLC17A5*	*slc17a5*	ihb263	ihb262
	Galactosialidosis, several forms	*PPGB*	*ctsa*	sa14856	
	Multiple sulfatase deficiency	*SUMF1*	*sumf1*	sa31531	
				la015919Tg	
**28. Osteolysis**	Mandibuloacral dysplasia	*ZMPSTE24*	*zmpste24*	sa9593	sa10379
				la013076Tg	
	Multicentric carpal-tarsal osteolysis with and without nephropathy	*MAFB*	*mafba*	b337*	uq4bh*
				nkgsaizgffm35aGt*	
		MAFB	*mafbb*	sa7349	
**29. Disorganized development of skeletal components group**	Cherubism	*SH3BP2*	*sh3bp2*	sa9540	sa11802
				sa14545	sa21687
				sa21688	sa34863
**30. Overgrowth (tall stature) syndromes with skeletal involvement**	Sotos syndrome/Marshall–Smith syndrome	*NFIX*	*nfixa*	sa19599	sa39695
				zko1017a	zko1017b
				la028183Tg	
			nfixb	sa10725	sa10966
				sa26010	sa40016
	Sotos syndrome	*NSD1*	*nsd1a*	sa13619	sa42452
				sa42453	la019080Tg
				la027572Tg	
			nsd1b	sa24006	sa30724
				sa37358	sa43693
				sa43694	
		APC2	*apc2*	sa4406	sa31814
				sa12808	sa41778
	CLOVES	*PIK3CA*	*pik3ca*	sa16936	sa25449
				sa35126	sa35127
				sa35128	sa45434

	Congenital contractural arachnodactyly	*FBN2*	*fbn2a*	sa14044	sa34870
				sa14555	sa34871
				sa17112	sa34872
				sa21695	sa34874
				sa21696	sa41614
				sa21697	sa41615
				sa27582	sa41617
				sa31772	
	Meester–Loeys syndrome	*BGN*	*bgna*	sa34346	
	Tall stature with long halluces, NPR3 type	*NPR3*	*npr3*	sa6997	la015689Tg
				la025074Tg	la029106Tg
**31. Genetic inflammatory/ rheumatoid-like osteoarthropathies**	CINCA / neonatal onset multisystem inflammatory disease (NOMID)	*CIAS1*	*nlrp3*	sa31246	sa32798
				sa32799	sa32801
	CRMO with CDA; Majeed syndrome	*LPIN2*	*lpin2*	sa18199	sa44534
**32. Cleidocranial dysplasia and related disorders**	Yunis–Varon dysplasia	*FIG4*	*fig4a*	sa10829	sa15712
				sa23824	sa29476
		VAC14	*vac14*	sa8773	sa38043
				la014679Tg	
**33. Craniosynostosis syndromes**	Antley–Bixler syndrome	*POR*	*pora*	sa4381	sa9088
				sa13459	sa21780
				sa38807	sa41702
			porb	sa39022	la027603Tg
	Baller–Gerold syndrome	*RECQL4*	*recql4*	sa15595	sa23411
				sa23412	sa25080
				sa32227	sa36752
	Carpenter syndrome	*RAB23*	*rab23*	hu2164	sa13880
**34. Dysostoses with predominant craniofacial involvement**	Miller syndrome (postaxial acrofacial dysostosis)	*DHODH*	*dhodh*	sa41042	
	Craniofrontonasal syndrome	*EFNB1*	*efnb1*	nim26*	nim25Tg*
				la029093Tg	hza102
	Acromelic frontonasal dysostosis	*ZSWIM6*	*zswim6*	sa31808	a365*
	Auriculocondylar syndrome, type 1	*GNAI3*	*gnai3*	sa21295	la026457Tg
	Auriculocondylar syndrome, type 2	*PLCB4*	*plcb4*	sa38208	
**35. Dysostoses with predominant vertebral with and without costal involvement**	NAD deficiency syndrome	*HAAO*	*haao*	vcc13	
		KYNU	*kynu*	vcc12	

	Klippel–Feil syndrome/Multiple synostoses syndrome (g.42)	*GDF6*	*gdf6b*	sa23521	sa36836
				sa43280	
	Cerebrocostomandibular syndrome (rib gap syndrome)	*SNRPB*	*snrpb*	sa31546	
**36. Patellar dysostoses**	Genitopatellar syndrome	*KAT6B*	*kat6b*	sa1676	sa14848
				sa15696	sa16535
				sa42204	
	Ear-patella-short stature syndrome (Meier–Gorlin)	*CDC45L*	*cdc45*	sa10592	la026603Tg
				la026601Tg	
**37. Brachydactylies (without extraskeletal manifestations)**	Brachydactyly with anonychia (Cooks syndrome)	*KCNJ2*	*kcnj2a*	ihb500	ihb372
			kcnj2b	ihb389	sa9367
				ihb388	
**38. Brachydactylies (with extraskeletal manifestations)**	Hyperphosphatasia with mental retardation, brachytelephalangy, and distinct face	*PIGV*	*pigv*	sa22895	sa45582
	Brachydactyly-hypertension syndrome (Bilginturan)	*PDE3A*	*pde3a*	(gt4Tg)*	(s892Tg)*
				(jh6Tg)*	
	Coffin–Siris syndrome	*ARID1B*	*arid1b*	sa8523	sa43376
				y607	
		SMARCB1	*smarcb1a*	la026498Tg	
		SMARCA4	*smarca4b*	sa24812	sa24813
				sa44439	
	Pseudohypoparathyroidism type IA	*GNAS*	*gnas*	sa9319	
**39. Limb hypoplasia–reduction defects**	Thrombocytopenia-absent radius (TAR)	*RBM8A*	*rbm8a*	oz36*	sa42812
	Adams-Oliver syndrome	*ARHGAP31*	*arhgap31*	sa20586	
		DOCK6	*dock6*	sa899	sa26161
				sa1010	sa33330
				sa6922	sa40178
				sa12512	
		EOGT	*eogt*	sa27753	sa38834
**40. Ectrodactyly with and without other manifestations**	Split-foot malformation with mesoaxial polydactyly (SFMMP)	*ZAK*	*map3k20a*	sa2499	sa20622
				sa8824	sa27328
				sa9459	sa33801
				sa20621	la026871Tg
**41. Polydactyly-Syndactyly-Triphalangism**	Cenani–Lenz like syndactyly/Oligosyndactyly, radio-ulnar synostosis, hearing loss and renal defects syndrome	*FMN1*	*fmn1*	sa1135	sa11299
				sa39286	sa43462
				la021518Tg	

	STAR syndrome (syndactyly of toes, telecanthus, ano- and renal malformations)	*FAM58A*	*ccnq*	sa11608	sa37673
				sa43955	
	Syndactyly with microcephaly and mental retardation (Filippi syndrome)	*CKAP2L*	*ckap2l*	sa40372	

Note: Genes in this table have not been associated by function or expression to skeletal development in available references. saXXXXX, fhXXX te(t)XXX, czXXX, huXXX, aXX and gwX are ENU-induced mutant strains from Stemple, Moens, Nüsslein-Volhard, Zon, Hubrecht Institute, Dowling and Gitlin Laboratories, respectively. laXXXXXXTg and hiXXXXTg are *Tg(nLacz-GTvirus)* transgenic strains from Burgess & Lin Lab and Hopkins Laboratory, respectively. zjuXXXTg is a *Tg(Xla.Eef1a1:RTTA-IRES-EGFP-5HS4,TETRE-CMV)* transgenic strain from Jun Chen Lab. itXXX, hzaXXX, zkoXX(X)b, tupX, zfXXXX, bnsXXX, tudXXTg, mkXX, nimXX, aXXX, unaX, yXXX, and ozXX are CRISPR1-edited mutants from Kobayashi, Ze-Xia Gao, China Zebrafish Resource Center (CZRC), Jao, Zhou, Stainier, Brand, Goll, Ober, Schier, Lomeli, Harold Burgess, and Amacher Labs., respectively. ihbXXX are CRISP or TALEN-edited mutants from Yonghua Sun lab. bnsXX, vccX are TALEN-edited mutants from Stainier and Kazu Kikuchi Lab. gtXXg is a *Tg(HSE:xx,HSE:GFP)* transgenic strain from Chong Shin Lab. nimXXTg is (*Tg2(5xUAS-E1B:xx,cryaa:Citrine)* transgenic strain from Ober Lab. sXXXTg is a *Tg(ins:CFP-NTR)* strain from Stainier Lab and jhXXg is a *Tg(ins:Kaede)* strain from Parsons Lab. Underlined strains can be found at ZIRC, EZRC, or CZRC as current source. Brackets is insertion site unclearly defined. Asterisk in genes indicates the paralogous studied. Asterisk in strains means studied but not related to a human skeletal disease.
